# Large-area epitaxial growth of curvature-stabilized ABC trilayer graphene

**DOI:** 10.1038/s41467-019-14022-3

**Published:** 2020-01-28

**Authors:** Zhaoli Gao, Sheng Wang, Joel Berry, Qicheng Zhang, Julian Gebhardt, William M. Parkin, Jose Avila, Hemian Yi, Chaoyu Chen, Sebastian Hurtado-Parra, Marija Drndić, Andrew M. Rappe, David J. Srolovitz, James M. Kikkawa, Zhengtang Luo, Maria C. Asensio, Feng Wang, A. T. Charlie Johnson

**Affiliations:** 1 Department of Physics and Astronomy, University of Pennsylvania, Philadelphia, PA 19104 USA; 2 Department of Biomedical Engineering, The Chinese University of Hong Kong, Shatin, Hong Kong; 3 Department of Physics, University of California at Berkeley, Berkeley, CA 94720 USA; 4 Department of Materials Science and Engineering, University of Pennsylvania, Philadelphia, PA 19104 USA; 5 Materials Science Division, Lawrence Livermore National Laboratory, Livermore, CA 94550 USA; 6 Department of Chemical and Biological Engineering, The Hong Kong University of Science and Technology, Clear Water Bay, Kowloon, Hong Kong; 7 Department of Chemistry, University of Pennsylvania, Philadelphia, PA 19104 USA; 8 Synchrotron-SOLEIL and Université Paris-Saclay Saint-Aubin, BP48, F91192 Gif sur Yvette Cedex, France; 9 Department of Materials Science and Engineering, City University of Hong Kong, Kowloon, Hong Kong; 10 Materials Science Institute of Madrid (ICMM), Spanish Scientific Research Council (CSIC), Valencia Institute of Materials Science (ICMUV), MATINÉE: CSIC Associated Unit-(ICMM-ICMUV Valencia University), E-28049 Cantoblanco, Madrid Spain; 11 Materials Science Division, Lawerence Berkeley National Laboratory, Berkeley, CA 94720 USA; 12 Berkeley and the Lawrence Berkeley National Laboratory, Kavli Energy NanoSciences Institute at the University of California, Berkeley, CA 94720 USA

**Keywords:** Synthesis of graphene, Synthesis of graphene, Structural properties, Two-dimensional materials

## Abstract

The properties of van der Waals (vdW) materials often vary dramatically with the atomic stacking order between layers, but this order can be difficult to control. Trilayer graphene (TLG) stacks in either a semimetallic ABA or a semiconducting ABC configuration with a gate-tunable band gap, but the latter has only been produced by exfoliation. Here we present a chemical vapor deposition approach to TLG growth that yields greatly enhanced fraction and size of ABC domains. The key insight is that substrate curvature can stabilize ABC domains. Controllable ABC yields ~59% were achieved by tailoring substrate curvature levels. ABC fractions remained high after transfer to device substrates, as confirmed by transport measurements revealing the expected tunable ABC band gap. Substrate topography engineering provides a path to large-scale synthesis of epitaxial ABC-TLG and other vdW materials.

## Abstract (web summary)

The semiconducting ABC configuration of trilayer graphene is more challenging to grow on large scales than its semimetallic ABA counterpart. Here, an approach to trilayer growth via chemical vapor deposition is presented that utilizes substrate curvature to yield enhanced fraction and size of ABC domains.

## Introduction

Van der Waals (vdW) materials composed of individual atomic layers held together by vdW interactions have attracted considerable attention due to their unique physical properties that can be tuned by manipulating interlayer twisting angles^1–3^, creating heterostructures^4,5^, and controlling stacking configurations^6,7^. One example is trilayer graphene (TLG)^8^, which has two stacking configurations in its natural form. The ABA or Bernal configuration is a semi-metal with tunable band overlap^9,10^, while the ABC or rhombohedral configuration is a semiconductor with a gate-tunable band gap^7,11,12^. In fact, few-layer graphene with ABC stacking is an interesting system for all thicknesses, as it is predicted to have topologically-protected surface states^13–15^ and high-temperature surface superconductivity^16,17^. Although ABA appears to be the ground state and is observed more often in exfoliated trilayer graphene, the thermodynamic preference over ABC is small, and a mixture is usually found. There are indications that the choice of substrate or the number of layers can affect this delicate ordering balance^18,19^.

To date, electronic transport studies of ABC-TLG have relied heavily on exfoliated samples, a process that is not scalable and limits the range of experimental investigations. A critical experimental challenge is to develop large-area synthesis of high-quality ABC-TLG^20–22^, which would catalyze a wide range of studies of the unexplored physics and applications of this unique material system. To answer this need, we develop a high-yield chemical vapor deposition (CVD) process to grow epitaxial TLG on Ni–Cu gradient alloy substrates. Angle resolved photoemission spectroscopy (ARPES) is used to confirm the epitaxial growth process and the characteristic band structures of ABC-TLG and ABA-TLG. Transmission electron microscopy is used to assess the atomic structure of the material, and it reveals the existence of quasi-lamellar patterns of ABA-TLG and ABC-TLG. Based on these observations, we develop a model for how substrate topography and, more specifically, the curvature of surface corrugations, plays a principal role in stabilizing ABC-TLG during growth. This curvature-based stacking selection (CBSS) mechanism is confirmed by an experiment correlating CVD-grown TLG material with the original growth substrate. We then demonstrate that the substrate curvature can be engineered through CVD growth time for controllable ABC-TLG yields of ~59%. We further find that transfer of TLG from the corrugated growth substrate to flat SiO_2_ leads to annihilation of ABA-ABC domain walls and increased ABC and ABA domain size, as revealed by infrared scanning near-field optical microscopy (IR-SNOM). Electron transport measurements demonstrate that the band gap of the CVD-grown ABC-TLG could be tuned by a perpendicular electric field. Our finding that substrate topography can be used to stabilize the ABC stacking structure may enable development of related CBSS synthesis strategies for other vdW epitaxies with specific interlayer stacking configurations and study concerning their physical properties and applications.

## Results

### Growth and characterization of ABC-TLG

**Fig. 1:**
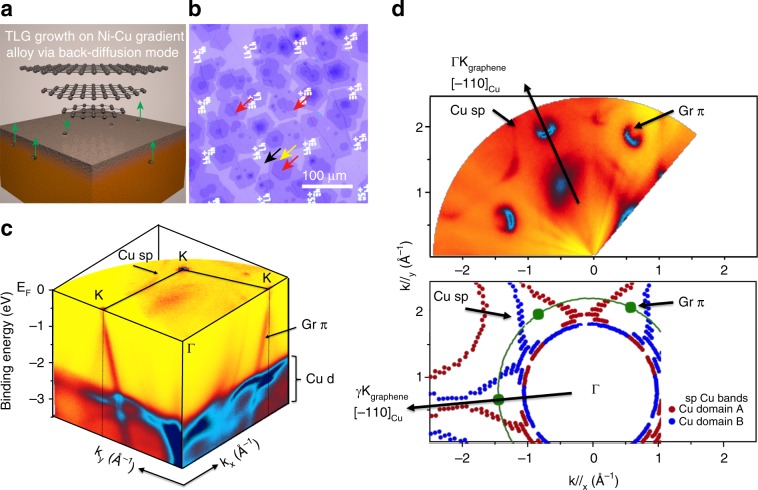
Epitaxial growth of TLG by CVD in back-diffusion mode. **a** Schematic of TLG growth on the Ni–Cu gradient alloy substrate via the carbon back-diffusion mechanism. Gray and orange represent Ni and Cu, respectively. **b** Optical micrograph of multilayer graphene formed on the Ni-rich side after 3 h of growth. For optical microscopy, the sample was transferred onto a 250 nm thick SiO_2_/Si substrate. The white regions are prefabricated alignment markers. The monolayer, bilayer, and trilayer regions are indicated by black, yellow, and red arrows respectively. The top monolayer graphene is a continuous film that spans the entire image, while the bilayer and trilayer regions remain hexagonal flakes. **c** 3D ARPES intensity plot showing the Fermi surface plane of the graphene film with electronic band structure of the graphene flakes and the metallic substrate. **d** Multi-grain Fermi surface of the samples showing graphene-substrate registry and the orientation of the grown graphene flakes. Top panel is the Fermi surface map. Bottom panel is the schematic diagram of the recorded Fermi surface, showing oriented graphene grains along the [$\bar 1$10] direction of the Cu (111) surface.

As shown schematically in Fig. 1a, TLG was synthesized on a Ni–Cu gradient alloy substrate in back-diffusion growth mode^23^. Catalytic substrates were prepared by depositing a 100-nm thick Ni film on one side of a flat Cu foil. The Ni–Cu foils were then annealed at 1050 °C for 5 min to create a Ni–Cu gradient alloy substrate with a Ni-rich side and a Ni-poor side. During CVD growth, the top TLG layer forms first over the Ni-rich side of the substrate, and then the middle and bottom TLG layers grow beneath the top layer via carbon back-diffusion from the Ni-poor side^23^. Additional information about the growth process is provided in the Methods section. As shown in Fig. 1b, the process resulted in high-yield TLG growth, with an areal coverage of ~30% over the entire substrate and flake sizes of 15–50 µm. All as-grown TLG domains displayed a compact hexagonal shape, attesting to their highly oriented, crystalline nature.

We performed systematic micro-ARPES and nano-ARPES measurements of the band dispersions of the material on the growth substrate, with 140 µm and 120 nm spot size respectively, to evaluate the electronic properties of the as-grown material. We directly probed the binding energy vs. momentum (*E* vs. $k_{//}$) curves close to the Fermi level; these are strikingly distinctive for TLG with ABA, ABC, and AAA stacking configurations^24^. The orientation, size, and distribution of graphene grains, as well as their registry with the substrate were investigated using micro-ARPES. Figure 1c shows a 3D ARPES plot, including the Fermi surface plane of the multi-grain graphene film, which discloses the complete electronic band structure of the graphene flakes and the metallic substrates. Strong π Dirac graphene bands are visible at the K high symmetry points of the reciprocal lattice. Moreover, well-defined *d* bands from Cu and/or Cu–Ni alloy are evident with binding energies of 2–4 eV. Distinctive *d* and *sp* substrate bands in the 3D ARPES plots indicate a high degree of crystallinity in the metallic substrate foils after CVD growth.

The graphene-substrate registry and the orientation of the graphene flakes are revealed by the multi-grain Fermi surface shown in Fig. 1d. A set of copper *sp* bands are observed dispersing in reciprocal space as expected for a copper single crystal (111) plane. Three well-defined Dirac cones are observed at $k_{//} =$ 1.703 Å^−1^, corresponding to the π graphene bands that cross the Fermi level. In contrast to earlier reports on few-layer graphene grown by CVD^24^, no arc-like shape states around the cones are observed, indicating that our graphene flakes are oriented along the ΓΚ_graphene_ [$\bar 1$10] direction of copper, in registry with the substrate lattice^25^. These results demonstrate that our process leads to epitaxial growth of TLG on the Ni–Cu gradient alloy substrate.

**Fig. 2:**
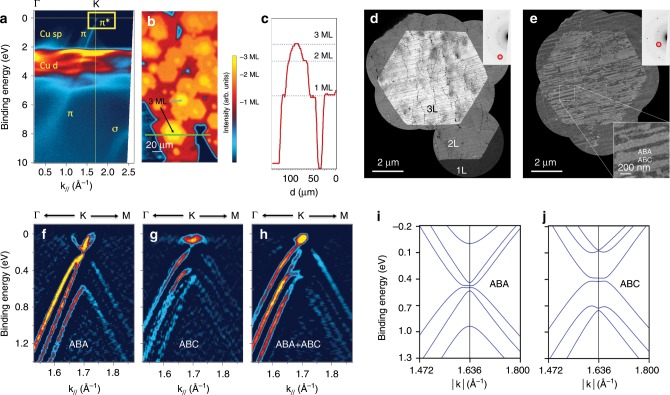
Electronic structures and stacking morphologies of TLG. **a** ARPES band structure of the graphene sample on the Ni–Cu substrate. The yellow rectangle indicates the energy-momentum region used for the intensity map in **b. b** Intensity map of electronic states using the energy-momentum region indicated in a. The black and blue arrows indicate ABA-TLG and ABC-TLG flakes, respectively. **c** Line scan profiles of the green line in panel **a**. **d** DFTEM image of a TLG flake with contrast between monolayer (dark gray), bilayer (light gray), and trilayer (white) regions. The inset shows the inverted selected area electron diffraction (SAED) pattern from the trilayer region, and the red circle indicates the $\left\{ {1\bar 210} \right\}$ spot selected to provide layer number contrast. The image was created by stitching together several smaller images; unimaged regions are left black. **e** DFTEM image with contrast between ABA (brighter) and ABC (darker) regions, as indicated in the lower inset. The upper inset shows the inverted SAED pattern from the trilayer region, and the red circle indicates the $\left\{ {0\bar 110} \right\}$ spot selected to provide stacking contrast. **f**–**h** ARPES band structure of **f**, ABA-TLG **g**, ABC-TLG and **h**, mixed-TLG. **i**–**j**, DFT band structures of **i**, ABA-TLG and **j**, ABC-TLG on Cu (111) in the vicinity of the K point (see also Supplementary Fig. 4). The energetic position of the occupied Cu bands is in line with previous calculations^55^ and the quantitative difference compared to experiments in Fig. 2a can be attributed to well-known shortcomings of conventional DFT methods, e.g., also observed for graphene on Ni^56, 57^.

The ARPES plot of Fig. 2a was used to select those states exclusively belonging to the graphene flakes and then record the nano-ARPES image of Fig. 2b. This image displays the intensity variations of the Dirac states at the Fermi level (yellow rectangle in Fig. 2a) throughout a large portion of the sample and reveals the distribution, size, and morphology of the graphene flakes. All the flakes exhibit identically-oriented hexagonal shapes, some more intense than the others in Fig. 2b (indicated by arrows, the intensity line scan profile of the green line is shown in Fig. 2c). To precisely and unequivocally resolve the electronic structure of each flake, the electronic band dispersion close to the Fermi level (*E* vs. momentum) was recorded for individual graphene grains using high energy and momentum resolution nano-ARPES^26^. The most intense flakes in Fig. 2b show TLG electronic dispersion characteristics; the flake indicated by the black arrow has two bands with quadratic dispersion and one band with linear dispersion (Fig. 2f), as expected for ABA-TLG. The flake indicated by the blue arrow has a cubic band that is particularly flat at the Fermi level and two quadratic bands at similar energies that are shifted along $k_{//}$. (Fig. 2g), as expected for ABC-TLG^27,28^. TLG flakes containing a mixture of both stacking types were also identified (Fig. 2h). All TLG flakes were n-doped, with the Dirac point located below the Fermi level due to the combined effects of the electrostatic surface potential and the “pillow effect” caused by surface adsorbates^29–31^. These nano-ARPES results are consistent with our density-functional theory (DFT) calculations (Fig. 2i–j, Supplementary Figs. 8, 9), which qualitatively confirm the observed band dispersions for the two TLG configurations, as well as the flat substrate bands around −1.5 eV (see Supplementary Fig. 4). Due to differences in the density of states around the Dirac point, the induced charge transfer from the substrate to graphene leads to a greater band shift in ABA-TLG as compared with ABC-TLG, in agreement with the data in Fig. 2f–g.

Dark-field transmission electron microscopy (DFTEM) was used to probe the stacking morphology and crystallinity of the as-grown TLG. This method can provide layer number contrast for oriented graphene^32^ (see Methods section for details). Figure 2d shows a DFTEM image generated by selecting one of the six $\left\{ {1\bar 210} \right\}$. diffracted beams with the objective aperture. For oriented TLG, these diffracted beams from each layer constructively interfere, and the contrast between monolayer, bilayer, and trilayer graphene is evident in the image. The uniform appearance of the trilayer hexagon in the middle suggests that the TLG is crystalline and highly oriented. To obtain contrast between different stacking configurations, we instead selected one of the six $\left\{ {0\bar 110} \right\}$ diffracted beams^33^, which with ABC stacking completely destructively interferes and with ABA stacking only partially interferes. The $\left\{ {0\bar 110} \right\}$ DFTEM image (Fig. 2e) of the same region shown in Fig. 2d reveals a quasi-lamellar pattern of TLG regions with ABA and ABC stackings. Approximately 30% of the TLG flake is in the ABC configuration, twice that reported for exfoliated samples^34^ and larger than usual levels in graphite. We observed flakes with up to 100% ABC content (see Supplementary Fig. 2), as also observed in exfoliated samples^35^. AAA stacking was not observed, consistent with the ARPES results, while some twisted TLG was obtained (see Supplementary Fig. 3).

### CBSS mechanism of ABC-TLG formation

The large-area fraction of ABC phase and the quasi-lamellar ABA/ABC domain morphology (Fig. 2e) suggest that the growth substrate properties and/or growth kinetics have an ABC-stabilizing effect. We performed ab initio calculations on flat, idealized substrates to first determine whether substrate chemistry contributes to this effect. We found that the relative energetics of multilayer graphene adsorbate/substrate systems are dominated by the interaction strength of the bottom-most graphene layer with the substrate, while differences due to variations in stacking of the upper layers are small. Energy differences computed between the most stable ABA and ABC arrangements on both Cu(111) and Ni-doped Cu(111) are therefore also small (~0.1 meV/atom) and barely within the accuracy of DFT (see Supplementary Note 1). We concluded that ABA and ABC are nearly isoenergetic on flat Cu(111) and Ni-doped Cu(111), in line with the coexistence of both structures in graphite and exfoliated TLG^34^. Importantly, this near equivalence between ABA and ABC energies suggests that the abundance of the two states can be tuned in finite-sized flakes by modulating other factors that slightly bias their relative energetics.

**Fig. 3:**
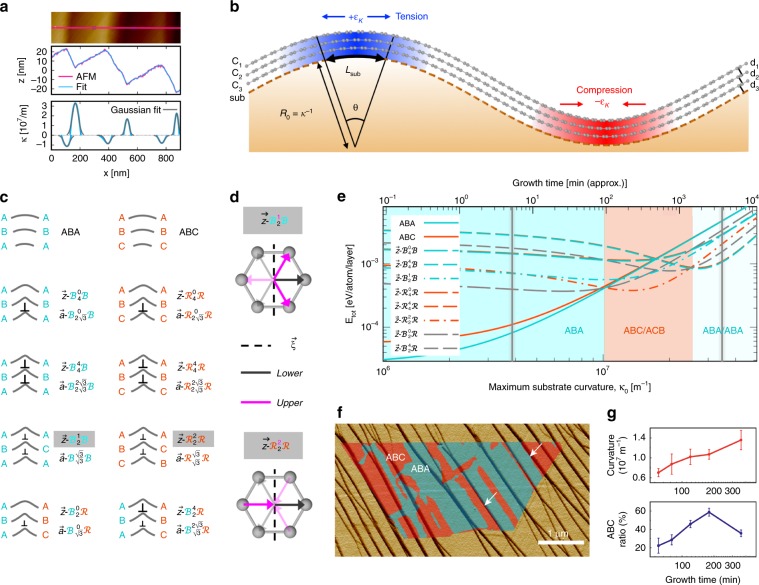
Curvature-stabilized ABC-TLG. **a** AFM topography image showing the corrugated substrate surface, 1D topography profile with a Fourier series fit, and Gaussian fits to the corresponding curvature profiles at corrugation extrema. The AFM image of the substrate was taken after growth and transfer of the graphene film. The measured curvatures were within the resolution of our AFM (see Supplementary Fig. 5). **b** Schematic of TLG on curved Cu demonstrating how geometric curvature and interlayer-interactions lead to in-plane strain, *ε*_*κ*_ . C_*i*_ denotes graphene where *i* is the layer index. Substrate and graphene curve lengths are *L*_sub_ $= R_0\theta$ and $L_i = [R_0 + \left( {4 - i} \right)d_i]\theta$, respectively, such that $\varepsilon _\kappa \approx (L_i - L_{i + 1})/L_i \approx d_i/R_0 = \kappa d_i$. **c** Schematics of interlayer dislocation configurations in TLG. $\bot$ and $\bot$ denote full and partial dislocations, respectively. The notation is described in the text. **d** Top views of the ${\mathbf{z}}-{\cal{B}}_2^1{\cal{B}}$. and ${\mathbf{z}}-{\cal{R}}_2^2{\cal{R}}$ interfaces, depicting the origin of CBSS for ABC-TLG; the larger Burgers vector edge component **b**_e_ (normal to the interface) in ABC/ACB domain walls. The three possible (**b**) directions for each interlayer are shown (arrows); those producing maximum strain relief (largest **b**_e_) are emphasized. **e** Computed energies *E*_tot_ of the states in **c** vs. maximum curvature *κ*_0_ for the zigzag dislocation line direction. Colored regions correspond to different equilibrium states, and vertical gray lines enclose the typical experimental range of *κ*_0_ values (the average was $\kappa _0 \approx 1.5 \times 10^7{\mathrm{m}}^{ - 1}$). **f** DFTEM map of ABA (turquoise) and ABC (red-orange) regions onto AFM amplitude image (yellow) of the corresponding growth substrate (see Supplementary Fig. 20 for DFTEM and AFM topography data). White arrows denote maximum curvature lines separating black and yellow regions. AFM amplitude: 348–353 mV. **g** Measured substrate corrugation peak curvatures and ABC ratios within TLG regions vs. CVD growth time. Error bars are the standard deviation.

One such factor is substrate topography, which we discovered can be exploited to energetically stabilize the (typically) less prevalent ABC phase. Our growth substrates had a quasi-periodic pattern of surface corrugations (discussed further below) with typical curvatures of $|\kappa _0| \approx 10^7{\mathrm{m}}^{ - 1}$ at maxima and minima (Fig. 3a). When adjacent graphene layers retain atomic registry, substrate curvature induces an in-plane interlayer strain $\varepsilon _\kappa (x) \approx \kappa (x)d \approx 0.5\%$, where *d* is the distance between graphene layers (Fig. 3b). Graphene must stretch (compress) in regions of negative (positive) curvature to maintain registry with adjacent layers. Above a critical curvature *κ*_c_, interlayer slip (i.e., interlayer dislocation formation) becomes energetically preferable to maintaining atomic registry between layers at all points. For weakly bound atomic layers of the type studied here^36^, *κ*_c_ is $\sim \!\!10^6-10^7{\mathrm{m}}^{ - 1}$. Growth on a corrugated substrate with *κ*_0_ > *κ*_c_ will result in arrays of interlayer dislocation lines, several types of which induce stacking shifts between ABA and ABC (i.e., domain walls = curvature-induced interlayer dislocations, see Fig. 3c and Supplementary Note 2).

To quantify these effects and identify the factors that control interlayer dislocation^37^ formation and domain size, we formulated a first principles-informed continuum mechanics description of the structure of vdW materials on non-flat surfaces (see Methods section for details). The dislocation character of a domain wall is denoted ${\mathbf{\xi }}-X_l^uY$, where ${\mathbf{\xi }}$ is the domain wall line direction (**z** = zigzag or **a** **=** armchair), *X* and *Y* specify the stacking configuration (${\cal{B}} \equiv$ Bernal/ABA, ${\cal{R}} \equiv$ rhombohedral/ABC) on the left and right side of the interface, respectively, and *u* and *l* specify the magnitude of **b**_e_, the edge component of the Burgers vector **b** (in units of *a*/2, where *a* = 0.14 nm is the atomic nearest neighbor distance) between the upper two and lower two graphene layers, respectively (see Fig. 3c and Supplementary Note 2 for further details of the notation).

The results demonstrated that curvature stabilizes interlayer dislocations and that certain types of stable dislocations in turn imply the presence of ABC domains. Specifically, our model indicates that the equilibrium state changes with increasing curvature from perfect ABA to states containing interlayer dislocations (Fig. 3e). A substrate with appropriate surface curvature (red-orange region in Fig. 3e) could fully stabilize arbitrarily large ABC domains containing only ${\mathbf{z}}-{\cal{R}}_2^2{\cal{R}}$ interfaces. However, the experimental values of *κ*_0_ span from ABA equilibrium (darker turquoise region) to ${\mathbf{z}}-{\cal{B}}_4^4{\cal{B}}$ equilibrium (lighter turquoise region), such that some flakes may be perfect ABA, some ABC/ACB (${\mathbf{z}}-{\cal{R}}_2^2{\cal{R}}$) or ABA/ACA (${\mathbf{z}}-{\cal{B}}_4^4{\cal{B}}$), and some a mixture of ABA and ABC. The computed energies of the ${\mathbf{z}}-{\cal{B}}_2^0{\cal{R}}$ (mixed ABA/ABC), perfect ABC, and ${\mathbf{z}}-{\cal{B}}_2^1{\cal{B}}$ (ABA/ACA) states are only slightly higher than those of the equilibrium states within the region of interest, indicating that these may also be observed. This is in agreement with the identification of pure ABA, pure ABC, and flakes with both stackings via nano-ARPES (Fig. 2f–h) and DFTEM (Fig. 2e and Supplementary Fig. 2). This stabilization of the ABC phase results from the difference in geometrically allowed partial-dislocation content between ABA and ABC stacking variants, as detailed in Supplementary Note 2 and Supplementary Figs. 10–14. Principally, ABC/ACB configurations (${\mathbf{z}}-{\cal{R}}_2^2{\cal{R}}$) accommodate 1D curvature strain more efficiently than ABA/ACA (${\mathbf{z}}-{\cal{B}}_2^1{\cal{B}}$) due to their larger edge character (larger **b**_e_, see Fig. 3d). The ABC phase is therefore preferred over an intermediate range of curvatures despite its larger bulk energy.

Our description of CBSS indicates that rich internal stacking structures will be commonplace in CVD multilayer graphene systems (Supplementary Fig. 25) and provides a framework for understanding how the interplay between corrugations and domain walls can stabilize different structures. The more prevalent views that assume internally structureless flakes (e.g., ABAB…-stacked multilayer graphene islands^38^) and/or structural domains with only simple periodic stacking sequences (e.g., a mixture of ABAB… and ABCABC…^33^) are not likely representative of actual states.

To directly test the CBSS mechanism, we marked a CVD-grown sample such that DFTEM images of domain wall morphologies could be mapped back to AFM images of the substrate topography at the precise location where growth occurred (see Supplementary Note 3 and Supplementary Fig. 19 for experimental details). Figure 3f shows that the dominant domain wall direction is precisely aligned with that of the substrate corrugations, and further that individual domain walls are strongly correlated with lines of maximum curvature (indicated by white arrows in Fig. 3f). Imperfect correlation is expected due to substrate corrugation irregularities and changes in domain wall configuration induced during cooling and transfer. Importantly, we found that substrate regions with lower than average corrugation curvatures (e.g., at grains with atypical surface crystal orientations) yielded significantly less ABC coverage than those with curvatures in or near the ABC/ACB-stabilizing range shown in Fig. 3e. This is seen clearly in the IR-SNOM stacking map (Supplementary Fig. 6) for a single TLG flake that spans three different Cu grains. The map shows 40% ABC coverage over one grain that has near-optimal curvature (~1–2 × 10^7^ m^−1^) and only 7% ABC coverage over the other two grains which have significantly lower curvatures (~1–2 × 10^6^ m^−1^). Further details of the IR-SNOM technique are provided below.

Previous studies have shown that ABC-TLG domain size can be manipulated using an electric field^39^ or mechanical force^40^ to drive domain wall motion. Here we have uncovered a new factor, substrate curvature, that governs ABC-TLG domain size and shape during synthesis. Substrate corrugations of the type seen here, long observed on graphene growth substrates but poorly understood, have recently been shown to form and coarsen beneath graphene *during* growth^41^. We propose (and provide evidence) that the primary driving force for this phenomenon is the reduction of the total step/curvature-induced interlayer disregistry/dislocation energy ($E_{{\mathrm{disreg}}} + E_{{\mathrm{disloc}}}$, see Methods section) as substrate step edges merge into increasingly coarse corrugations (see Supplementary Note 5 and Supplementary Fig. 22). The resulting corrugation evolution during growth feeds back into graphene stacking domain morphology in a co-evolving process. Incorporating this overlayer-driven corrugation kinetics into our continuum model, we obtain a time-dependent description of domain wall energetics during simultaneous corrugation coarsening and graphene growth (see Supplementary Note 5 and Fig. 3e).

These insights indicate that ABC yield can be controlled and optimized by engineering curvature through tailoring synthesis parameters (growth time, temperature, substrate chemistry, crystallography). Accordingly, increasing growth time from 1 to 6 h led to a monotonic increase in the final average peak corrugation curvatures (measured on our Cu (111) substrates) from ~7.0 × 10^6^ to ~1.4 × 10^7^ m^−1^ (Fig. 3g and Supplementary Fig. 23). ABC yield also increased monotonically from ~22% to ~59% between 1 and 3 h, before decreasing to ~36% at 6 h (Fig. 3g and Supplementary Fig. 24). The increase in ABC yield to a maximum at substrate curvatures ~1.0–1.2 × 10^7^ m^−1^ is in good agreement with the predictions of Fig. 3e, and the decrease at 6 h is consistent with our expectation of a finite range of optimal curvatures. These results substantiate our physical description of CBSS and demonstrate that the growth process can be designed to control and optimize ABC yield. More precise control and higher yields may be achievable with further exploration of the effects of growth temperature, substrate chemistry, and substrate crystallography (see Supplementary Note 5).

**Fig. 4:**
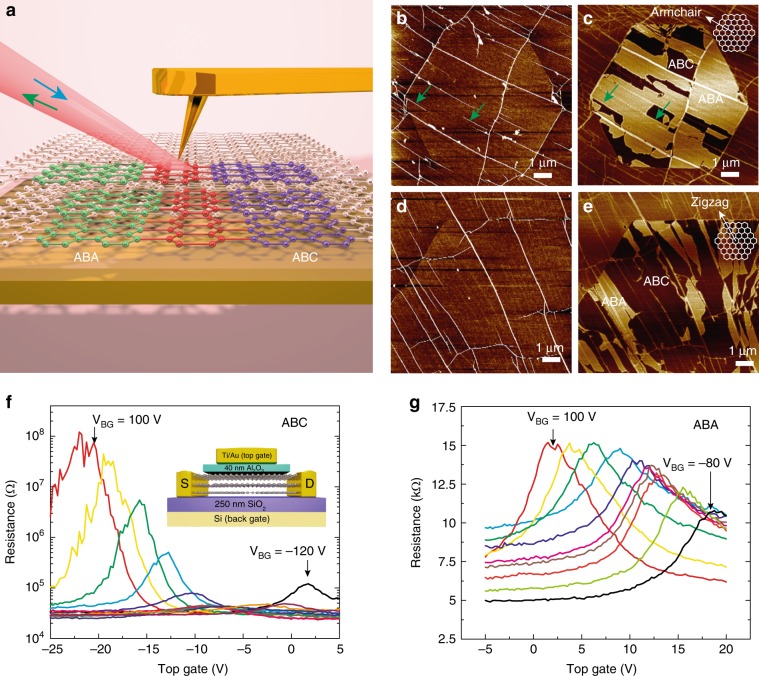
IR-SNOM and electron transport characterization of ABC-TLG and ABA-TLG. **a** Schematic of IR-SNOM for characterization of ABC-TLG on a SiO_2_ substrate. **b**, **d** AFM topography image of TLG flakes showing uniform heights across the entire flake except along wrinkles (white lines). The height scale is 2 nm. **c**, **e** Corresponding IR-SNOM image of the TLG flake in **b** and **d**, ABA-TLG and ABC-TLG show different infrared contrasts, which are absent in the topography image. **c** has primarily armchair domain walls, and **e** has primarily zigzag domain walls. (contrast scale: 1.43 V in **c** and 2.20 V in **e**). Green arrows in **c** indicate correlated wrinkles and ABA-ABC domain walls. **f** *R*–*V*_TG_ characteristics of CVD ABC-TLG at 1.8 K. Each curve was measured with a fixed *V*_BG_ ranging from −120 V to 100 V (20 V steps). The on/off ratio increases with *V*_BG_ due to the band gap opening of ABC-TLG. The inset shows a schematic of the dual-gated TLG device. **g** *R*–*V*_TG_ characteristics of CVD ABA-TLG at 1.8 K. Each curve was measured with a fixed *V*_BG_ ranging from −80 V to 100 V (20 V steps). The net doping (n-type or p-type) varied from device to device^42^, depending on details of the fabrication process in which charged impurities are introduced.

We performed additional experiments to confirm that curvature-stabilized ABC domains were preserved and exhibited appropriate electronic properties after transfer and conformation to flat dielectric substrates commonly used for device fabrication. TLG flakes were transferred to a SiO_2_ substrate and imaged with infrared scanning near-field optical microscopy (IR-SNOM; Fig. 4a). In this measurement, infrared light (red beam in the figure) is focused onto a metal-coated AFM tip, and the backscattered light is collected by a detector in the far field. Local infrared conductivities are thus obtained across the TLG flake, generating contrast between ABA (green) and ABC (purple) regions^40,42^. IR-SNOM is uniquely suited for the task of analyzing the domain structure of the TLG synthesized here because its tip-enhanced signal offers ~20 nm spatial resolution (diameter of the metallic AFM tip), greatly superior to the ~1 µm typically achieved by Raman spectroscopy^34^.

Figures 4b, d show TLG flake topographies as measured by AFM. The flake height is uniform across the hexagonal domains, except at the 3–7 nm high wrinkles visible as white lines. The corresponding IR-SNOM image, however, exhibits distinct contrast between ABA and ABC regions (Fig. 4c, e). Both ABA-TLG and ABC-TLG were observed via IR-SNOM after transfer to SiO_2_, and three striking phenomena were identified. (1) Quasi-lamellar stacking domains, similar to the DFTEM image of Fig. 2e, were observed. (2) ABA and ABC regions identified by IR-SNOM were ~10 times wider (~1–2 μm) on average than those seen by DFTEM, with significantly reduced domain wall density (compare Fig. 2e with Fig. 4c, e). (3) Wrinkles and ABA-ABC domain walls were highly correlated (see green arrows in Fig. 4b, c). TLG flakes with armchair (zigzag) domain wall line directions exhibited an average ABC coverage of 28% (59%) and an average area of 1.4 µm^2^ (5.6 µm^2^) (see Supplementary Fig. 7).

These phenomena can be understood in light of the physical picture described by our continuum model. First, the difference in ABC-TLG yield between samples with zigzag and armchair domain walls is consistent with the prediction of our continuum model that maximum ABC stabilization occurs at the zigzag direction (see Fig. 3e and Supplementary Fig. 10). Second, the growth substrate possesses a quasi-periodic array of peaks and valleys with nearly equal but opposite curvature (Fig. 3a), which favors the creation of TLG with a quasi-periodic array of domain walls with alternating sign. After removal of the domain wall-stabilizing Ni–Cu substrate and transfer to flat SiO_2_, many adjacent interlayer dislocation pairs will be attracted toward each other, and those pairs whose Burgers vectors sum to zero will fully annihilate upon contact, as described in Supplementary Figs. 15–18. The average ABA and ABC domain sizes are therefore expected to increase upon transfer to flat SiO_2_. Strikingly, we found that large-area CVD ABC-TLG can be reliably retained after transfer onto an hBN substrate (Supplementary Fig. 26). This stands in sharp contrast to the case for exfoliated graphene, where it is reported^43^ that ABC-TLG is almost always converted to ABA-TLG after transfer onto hBN. Our CVD approach is therefore expected to have a significant impact on research directed at the Mott-insulator state^43^ and tunable superconductivity^44^ in TLG.

### Transport measurement of ABC-TLG

The large ABC-TLG and ABA-TLG domain sizes on SiO_2_ as identified by IR-SNOM allowed us to fabricate FET devices and perform transport measurements on TLG regions with different stacking configurations. Confirmation of their expected electronic behavior is critical for potential applications. As illustrated in the inset of Fig. 4f, FET devices were created in dual-gate configurations using 40 nm thick Al_2_O_3_ dielectric layers deposited by atomic layer deposition as the top-gate dielectric and 250 nm thick thermally grown SiO_2_ as the bottom-gate dielectric. We measured the resistance of TLG channels as a function of the top and bottom-gate voltages at a temperature of 1.8 K. Figure 4f shows resistance vs. top-gate voltage (*R*–*V*_TG_) curves of an ABC-TLG channel. The existence of a tunable energy band gap in the material is evidenced by the increase in the on/off ratio as the strength of the out-of-plane electric field is increased. The same measurement showed that a sizeable ABC band gap was also achieved at room temperature (Supplementary Note 4 and Supplementary Fig. 21), further evidence of the high quality of our epitaxially-grown TLG. At 1.8 K, an on/off ratio of more than 3000 was observed, comparable to exfoliated samples^35,45,46^. No such increase was observed for the ABA FET (Fig. 4g), in good agreement with its predicted band structure and measurements by others on exfoliated samples^7,45^.

## Discussion

We have demonstrated a scalable and controllable approach to CVD growth of TLG with an enhanced yield of large ABC-stacked domains. The growth strategy was based on a Ni–Cu gradient alloy substrate that facilitates the back-diffusion growth mode. The key mechanism introduced and underlying the approach is curvature-based stacking selection (CBSS); the use of substrate curvature on the nanoscale to generate interlayer dislocations and locally stabilize ABC domains. The quasi-periodic corrugated topography of our growth substrate (commonly observed) typically generates alternating lamellar regions of ABC and ABA, as revealed by DFTEM and IR-SNOM measurements. An optimal range of curvatures, tunable through growth time, leads to maximized ABC yield, consistent with our continuum CBSS model predictions. The coarsening of substrate topography beneath growing graphene overlayers is attributed to the reduction of interlayer disregistry/dislocation energy achieved by increasing corrugation scale. ARPES results on as-grown TLG confirmed the epitaxial nature and electronic hallmark of ABC-TLG and ABA-TLG, while the existence of a sizable, gate-tunable band gap for ABC-TLG regions was confirmed by electron transport measurements. Significant increases in ABC and ABA domain size were observed following transfer from growth substrates to flat SiO_2_ substrates, consistent with the expectation that interlayer dislocation pairs annihilate upon transfer to flat SiO_2_. CBSS, optimized through substrate topography engineering, is broadly applicable to epitaxial synthesis of vdW materials beyond ABC-TLG, and should enable further exploration of their promising physical properties and provide opportunities for applications in next-generation nanoelectronic devices.

## Methods

### TLG synthesis and transfer

The TLG growth was carried out in an atmospheric pressure CVD system with a 1-inch furnace (Lindberg Blue M, Thermo Scientific Co.). Cu foils of 25 µm thickness (Item #46365, Alfa Aesar) were cleaned with 5.4% HNO_3_ for 40 s and two DI water baths for 2 min each. A nickel film of 100 nm thickness was sputtered onto one side of the Cu foil (Lesker PVD75 DC/RF Sputter System). Ni–Cu foils were cut into 1 cm × 4 cm pieces and then loaded into the CVD furnace. The furnace was ramped to 1050 °C at a rate of 60 °C/min in a flow of 500 sccm Ar without H_2_. The growth substrate was annealed for 5 min at 1050 °C in a flow of 500 sccm Ar + 30 sccm H_2_. TLG was grown using 1.8 sccm CH_4_ (1% in Ar) + 30 sccm H_2_ for 3 h. The reactor was then rapidly cooled to room temperature in a flow of 10 sccm H_2_ and 1000 sccm Ar.

PMMA (MicroChem Corp., PMMA950, A4) was spun over the surface of the TLG as grown on Ni–Cu foils and then baked at 100 °C for 2 min The substrate was removed by an etcher solution (Transene Company, Inc. CE-100). The PMMA supported TLG was cleaned in 10% HCl solution and two water baths, and was transferred onto a 250 nm SiO_2_/Si substrate with prefabricated gold makers. After air drying and baking at 150 °C for 3 min, PMMA was removed by soaking the sample in acetone overnight and cleaning with IPA. TLG on SiO_2_/Si substrates were annealed in H_2_/Ar forming gas at 400 °C for 1 h before IR-SNOM characterization.

### IR-SNOM

Scattering-type scanning near-field optical microscopy is based on tapping mode atomic force microscopy (AFM). A metallic AFM tip is illuminated with a focused CO_2_ laser beam with wavelength 10.6 µm and the enhanced local field under the tip interacts strongly with the sample underneath it. The elastically scattered light containing the local optical properties of the sample substrate is collected by a MCT (HgCdTe) detector in the far field. Near-field optical images with spatial resolution of sub 20 nm can be achieved simultaneously with topography by recording the scattered light while scanning the sample. ABA/ABC graphene have different optical conductivities at 10.6 µm due to their different band structures and thus can be differentiated in the near-field images. In bilayer graphene, the contrast of domain walls in near-field images stems from surface plasmon reflection at the domain walls and feature a single or double bright line depending on the domain wall type^47^. Operation in tapping mode rather than contact mode^40^ prevents imaging-induced domain wall motion, and no such motion was observed in these experiments.

### AFM

An atomic force microscope (AFM, Icon Bruker) equipped with a probe with a tip radius of 10 nm (HQ-300-Au, Oxford Instruments) was used to characterize the topography of the growth substrate, in a different facility from that in which IR-SNOM measurements were performed.

### Fabrication of dual-gate TLG FET structures

ABA-TLG and ABC-TLG regions were first identified by IR-SNOM. ABA-TLG and ABA-TLG channels were then defined by e-beam lithography and oxygen plasma etching (Pressure: 1.25 Torr, Power: 50 W, Duration: 35 s). The remaining e-beam resist (PMMA950 C4, MicroChem Corp.) was removed using acetone and isopropanol (IPA). The top contact metallization for the FET device (5 nm Cr/40 nm Au) was deposited by e-beam lithography followed by thermal evaporation. After liftoff process, the device was annealed at 225 °C in the forming gas of 1000 sccm Ar + 250 sccm H_2_ for 1 h to reduce the PMMA residuals on TLG channels. The devices were spin-coated with a HSQ buffer layer (1% in MIBK, 6000 rpm for 60 s, softback at 80 °C for 4 min), followed by atomic layer deposition (Cambridge Nanotech S200) of 40 nm Al_2_O_3_ as the top-gate dielectric. Lastly, e-beam lithography was carried out to deposit 5 nm Ti/40 nm Au top-gates on the Al_2_O_3_ dielectric to form the dual-gate FET devices.

### Electrical transport measurement

Low temperature electrical transport measurements were performed in a Quantum Design Physical Property Measurement System at an ambient He pressure of ~5 Torr. Transport measurements were recorded using the drain as a common ground for the source and gates. Voltages for top and back gates were independently controlled by Keithley 2410 and Keithley 237 voltage sources, respectively. Gate leakage was verified to be <0.5 nA. Source-drain were biased for constant voltage using a second Keithley 237, with maximum power <0.5 nW across all measurements. After verifying source-drain linearity, source-drain current-voltage data with mV scale source bias was used to determine the channel resistance for each gate configuration. Each *R*–*V*_TG_ curve was measured with a fixed back-gate voltage (*V*_BG_) in steps of 20 V, and *V*_TG_ was swept continuously.

### DFTEM

TLG was transferred onto a Cu grid with an amorphous carbon support. An aperture was placed in the diffraction plane of an electron microscope to create a real space image from electrons scattered only at a specified angle. With oriented stacked graphene, the $\left\{ {1\bar 210} \right\}$ planes of A, B, and C layers are aligned, and the diffracted beams from each layer interfere constructively. Selecting one of the six $\{ 1\bar 210\}$ diffraction spots with the objective aperture give layer number contrast for oriented graphene. In ABC-TLG, the first order $\left\{ {0\bar 110} \right\}$ planes of B and C layers are displaced by 1/3 and 2/3 of the plane spacing, respectively, from the same planes in the A layer. The $\left\{ {0\bar 110} \right\}$ diffracted beams from each layer therefore show complete destructive interference in ABC-TLG. In ABA-TLG there is only partial destructive interference of the $\left\{ {0\bar 110} \right\}$ beams^33^. The $\left\{ {0\bar 110} \right\}$ diffraction spots therefore provide the desired stacking contrast in oriented TLG. In BLG, when selecting a $\left\{ {0\bar 110} \right\}$ diffracted beam, a non-zero sample tilt can break the symmetry between the AB and BA layers^32^. This can be seen in Fig. 2e, where although the layer normal of the sample was aligned to the optic axis, the not perfectly flat TEM support leads to contrast between AB and BA stackings in some areas of the bilayer region.

### ARPES

The ARPES and nano-ARPES experiments were carried out at the ANTARES beamline of synchrotron SOLEIL. It is equipped with a Fresnel zone plate (FZP) to focalize the beam and order selection aperture (OSA) to eliminate higher diffraction orders. The sample was mounted on a nano-positioning stage which was placed at the coincident focus point of the electron analyzer and the FZP. Nano-ARPES can be operated in two ways: (1) the point mode where a spectrum is collected from a specific point of the sample and (2) the imaging mode, where photoelectron intensity from a specific electron energy range is mapped over the sample to create a two dimensional image of the electronic states of interest. All photoemission measurements were performed at a temperature of ∼60 K. The photoelectron spectra were obtained using a hemispherical MBS analyzer. The instrumental energy resolution is ∼12 meV and practical momentum resolution is ∼0.02 1/Å for an emission angle ∼20°

### DFT calculations

All-electron density-functional theory (DFT) calculations were carried out with the Fritz-Haber-Institute-Ab initio-Molecular-Simulations^48^ package using the Perdew-Burke-Ernzerhof (PBE) generalized-gradient approximation^49^ supplemented by the TS method^50^ to account for dispersive interactions. We use the suggested tight setup and accompanied basis functions throughout. A Cu (111) surface was generated from an optimized copper slab using six layers, where the bottom three layers were kept fixed to their bulk positions. A vacuum layer of 30 Å was introduced to separate periodic images along z, and a finite-size correction for the slab was employed. The Brillouin zone was sampled by a 11 × 11 × 1 Monkhorst-Pack **k**-point grid^51^, and k-point convergence was improved by filling the electronic states with a Gaussian broadening of 0.1 eV. Models including nickel doping of 3.7% were calculated in 3 × 3 surface supercells of the above-described copper surface, reducing the number of layers to three and replacing one copper atom with nickel. Binding energies were defined throughout as $E_{\mathrm{b}} = E_0\left( {{\mathrm{Cu}}/n - {\mathrm{graphene}}} \right)-E_0\left( {{\mathrm{Cu}}} \right)-E_0\left( {n - {\mathrm{graphene}}} \right),$ where $E_0\left( {{\mathrm{Cu}}/n - {\mathrm{graphene}}} \right)$, *E*_0_(Cu), and $E_0\left( {n - {\mathrm{graphene}}} \right)$ are the total energies of the combined Cu(111) plus *n*-graphene sheet system, the defined Cu(111) surface, and *n*-graphene sheets, respectively. Transition states were searched with the climbing-image nudged-elastic-band method using five images.^52^

### Continuum model for CBSS in vdW materials

The total energy of an elastic multilayer system (weakly bonded sheets) was divided into contributions from in-plane disregistry strain, interlayer dislocations, and stacking phase bulk energies; $E_{{\mathrm{tot}}} = E_{{\mathrm{disreg}}} + E_{{\mathrm{disloc}}} + E_{{\mathrm{phase}}}$. For trilayer graphene on corrugated Cu (111), the substrate topography was assumed to vary only along *x* such that the disregistry energy over a region of size *L*_Cu_ (per unit length *y*) is

1 $$E_{{\mathrm{disreg}}} = \frac{{E^{2{\mathrm{D}}}}}{2}\mathop {\sum }\limits_{i = 1}^3 {\smallint }_0^{L_{{\mathrm{Cu}}}} \left[ {\varepsilon _i + \varepsilon _{\kappa ,i}\left( x \right)} \right]^2dx$$

where $\varepsilon _i = b_iN_i - \varepsilon _{0,i}$ is the elastic strain and $\varepsilon _{\kappa ,i}\left( x \right) = d_i/[\kappa ^{ - 1}\left( x \right) + {\sum }_{j = i + 1}^3 d_j]$ is the geometric strain. $E^{2{\mathrm{D}}} = 340\, {\mathrm{N}}/{\mathrm{m}}$^53^ is the 2D Young’s modulus of graphene, *i* is the interlayer index (*i* *=* 1, 2, and 3 correspond to C_1_–C_2_, C_2_–C_3_, and C_3_–Cu, respectively; see Fig. 3b), *b*_*i*_ is the dislocation Burgers vector magnitude along *x* (edge component), *N*_*i*_ is the integer dislocation density between *x* = 0 and *x* = *L*_Cu_, *ε*_0*,i*_ is the in-plane misfit strain between flat layers in *i*, and *d*_*i*_ = 0.335 nm^54^ is the interlayer separation. We separate geometric strains from elastic strains in $E_{{\mathrm{disreg}}}$ to emphasize the physical competition between these two effects. Geometric strain *ε*_*κ.i*_ is that introduced via curvature under the condition of epitaxial registry between layers (as illustrated in Fig. 3b), and elastic strain *ε*_*i*_ is that introduced by interlayer dislocations. Without dislocations, the entire geometric strain is stored as elastic energy within the system. With a particular amount of dislocations, the introduced elastic strain exactly offsets the geometric strain, and the strain energy goes to zero. With this form of $E_{{\mathrm{disreg}}}$, the strain reference state is that in which a given graphene layer rests strain-free on either the substrate (*i* = 3) or the graphene layer below it (*i* = 1 or 2). This form also invokes the assumption that dislocations relieve strain uniformly over each local peak or valley. The only non-material-parameter input into the model, the substrate curvature profile *κ*(*x*), was determined by fitting AFM-measured substrate topographies to a Fourier sine series and parameterizing the resulting curvatures at each corrugation extremum as $\kappa \left( x \right) = \kappa _0e^{ - x^2/2\sigma ^2}$ (Fig. 3a). We further employed $\varepsilon _{0,1} = \varepsilon _{0,2} = 0$, and neglected *i* *=* *3* (the substrate). Bending energy differences between states can also be neglected (the bending modulus of graphene is extremely small). The total dislocation line energy (per unit length *y*, adapted from ref. ^37^) was expressed as

2 $$E_{{\mathrm{disloc}}} = \mathop {\sum }\limits_{i = 1}^3 L_{{\mathrm{Cu}}}N_i\left[ {\frac{{b_i}}{a}\left( {E_{{\mathrm{edge}}}{\mathrm{sin}}^2\theta _i + E_{{\mathrm{screw}}}{\mathrm{cos}}^2\theta _i} \right) + \frac{{2b_i^2}}{{L_{{\mathrm{Cu}}}}}\left( {\frac{{E^{2{\mathrm{D}}}}}{4} + \frac{B}{{d_i^2}}} \right)} \right]$$

where *a* = 0.14 nm is the nearest neighbor distance in graphene, *E*_edge_ = 0.318 × 10^−10^ J/m and *E*_screw_ = 1.091 × 10^−10^ J/m are the core energy coefficients, $\theta _i$ is the angle between the dislocation line and Burgers vector directions, and *B* = 22.08 × 10^−20^ J is the bending modulus of graphene. The first term in the square brackets is the core energy and the second term is the contribution from long-range interactions between interlayer dislocations. The bulk energy of the trilayer is $E_{{\mathrm{phase}}} = E_iL_{{\mathrm{Cu}}}{\sum }_{j = 1}^3 d_j$ for single phase states, where *E*_*i*_ is the bulk energy of ABA or ABC per unit volume, and $E_{{\mathrm{phase}}} = \frac{1}{2}(E_{{\mathrm{ABA}}} + E_{{\mathrm{ABC}}})L_{{\mathrm{Cu}}}{\sum }_{j = 1}^3 d_j$ for two phase states.

We considered ten possible TLG states; perfect ABA, perfect ABC, and eight configurations with periodic dislocation arrays (Fig. 3c). There are five configuration categories: ABA-ABA, ABA-ACA, ABC-ABC, ABC-ACB, and ABA-ABC. The existence of the latter three was experimentally confirmed (see Supplementary Fig. 1; ABA and ACA cannot be distinguished by DFTEM^32^). The energy of each state was determined as a function of maximum local curvature κ_0_ (at σ = 16 nm, the average measured feature width), as shown in Fig. 3e for interlayer dislocations along the zigzag direction (see Supplementary Fig. 10 for the armchair direction).

## Supplementary information

Supplementary Information

## Data Availability

The data that support the findings of this study are available within the paper and its Supplementary Information, and all data are available from the corresponding authors upon reasonable request.
