# Supplementary material for: Large-area epitaxial growth of curvature-stabilized ABC trilayer graphene
Source: Nat Commun. 2020 Jan 28;11:546. doi: 10.1038/s41467-019-14022-3 (PMC6987307; doi:10.1038/s41467-019-14022-3)
Supplement: Supplementary file 1 — Supplementary Information [file 41467_2019_14022_MOESM1_ESM.pdf]

SUPPLEMENTARY INFORMATION

Large-Area Epitaxial Growth of Curvature-Stabilized ABC Trilayer Graphene

Gao et al.

Supplementary Figures

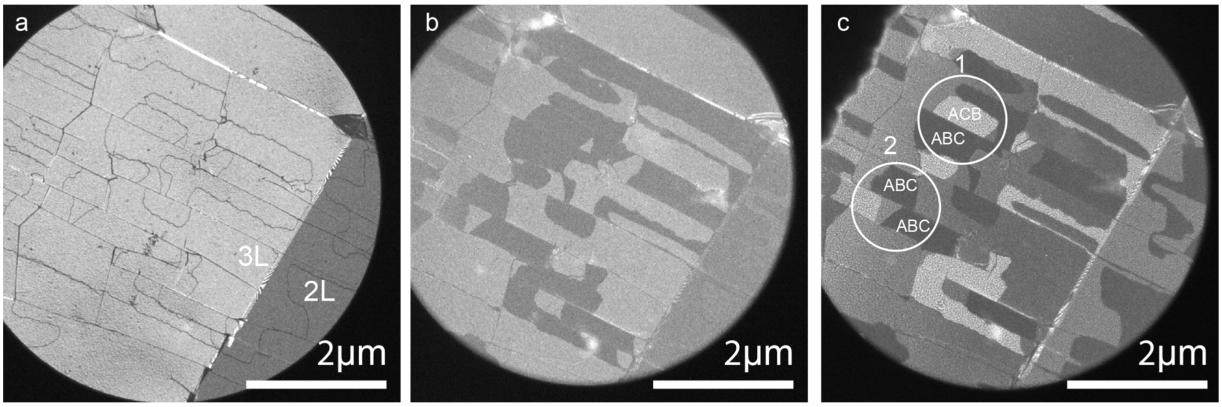

Supplementary Figure 1. DFTEM images of trilayer graphene (TLG) with ABC-ACB and ABC-ABC domain walls. **a**, Dark field transmission electron microscopy (DFTEM) image of a TLG flake with contrast between bilayer (dark gray), and trilayer (light gray) regions by selecting the $\{1\bar{2}10\}$ diffraction spot. **b**, DFTEM image with contrast between Bernal ABA (brighter) and rhombohedral ABC (darker) regions by selecting the $\{0\bar{1}10\}$ diffraction spot without sample tilting. **c**, $\{0\bar{1}10\}$ DFTEM image of a slightly tilted sample, where ABC- and ACB-TLG show different intensities due to the broken symmetry. Region 1 shows an ABC-ACB boundary and region 2 shows an ABC-ABC boundary. Note that the difference between ABA-TLG and ACA-TLG cannot be distinguished by DFTEM[1].

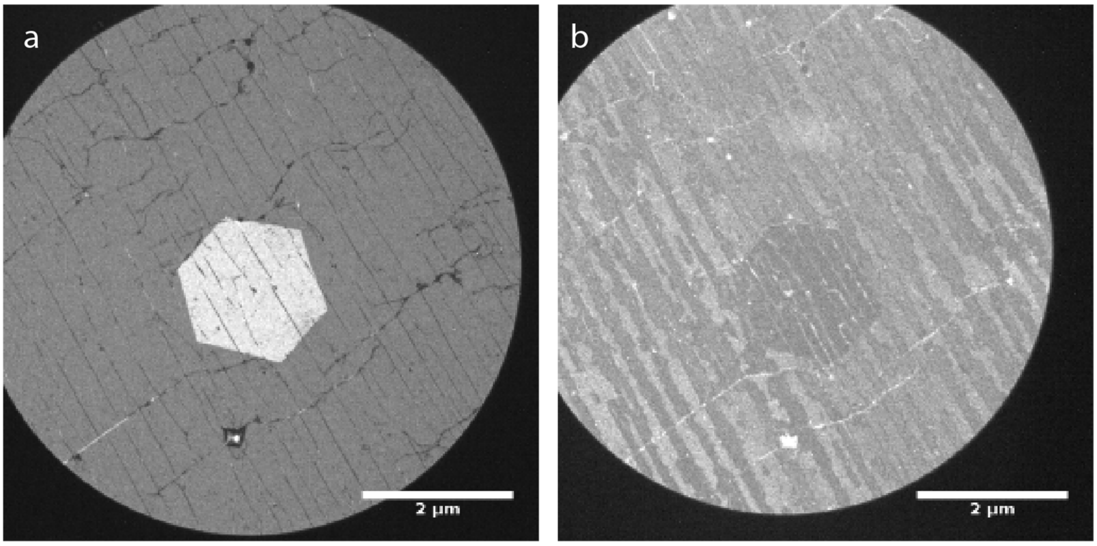

Supplementary Figure 2. DFTEM images of a TLG flake with 100% ABC stacking. **a**, The $\{1\bar{2}10\}$ diffraction spot produces contrast between bilayer (dark gray) and trilayer (light gray) regions. **b**, The $\{0\bar{1}10\}$ diffraction spot produces contrast between ABA and ABC regions, but no ABC-ABA contrast is observed within the TLG flake, indicating 100% ABC stacking.

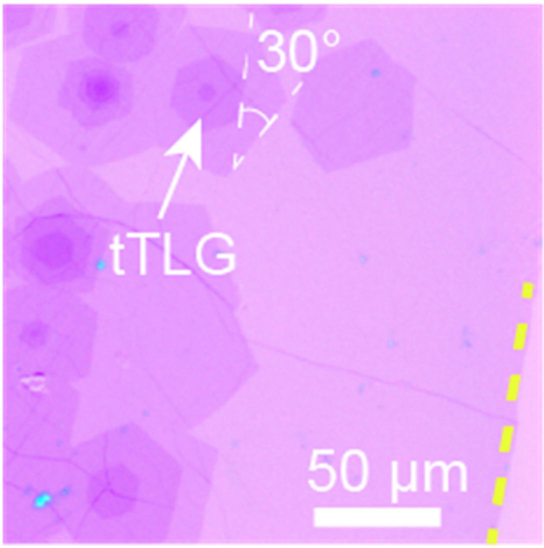

Supplementary Figure 3. Optical image of twisted trilayer graphene (tTLG) domains showing a preferred twist angle of ~30°. The orientation of the monolayer region is indicated by the dashed yellow line along its edge (bottom right).

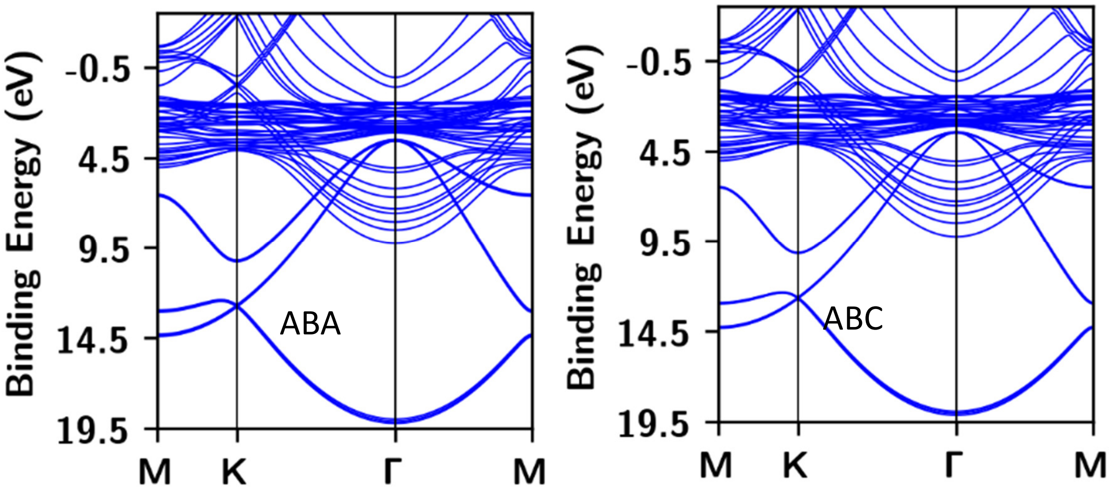

Supplementary Figure 4. Full DFT band structures of ABA- and ABC-TLG. Regions in the vicinity of the K points are shown in **Figs. 2i** and **2j**.

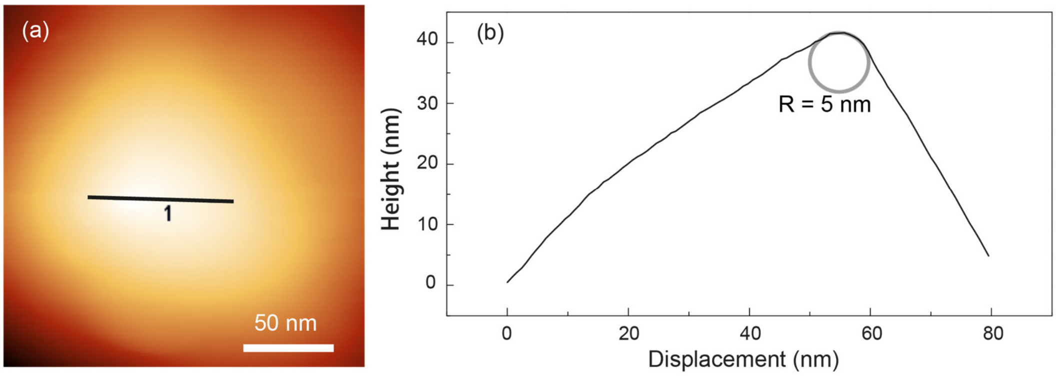

Supplementary Figure 5. Investigation of AFM resolution using a test grating tip (TGT1, NT-MDT) with a known curvature radius of ⩽10 nm. **a**, AFM image of the test grating tip. **b**, Line scan profile for the black line in **a**, indicating a tip curvature radius of 5 nm.

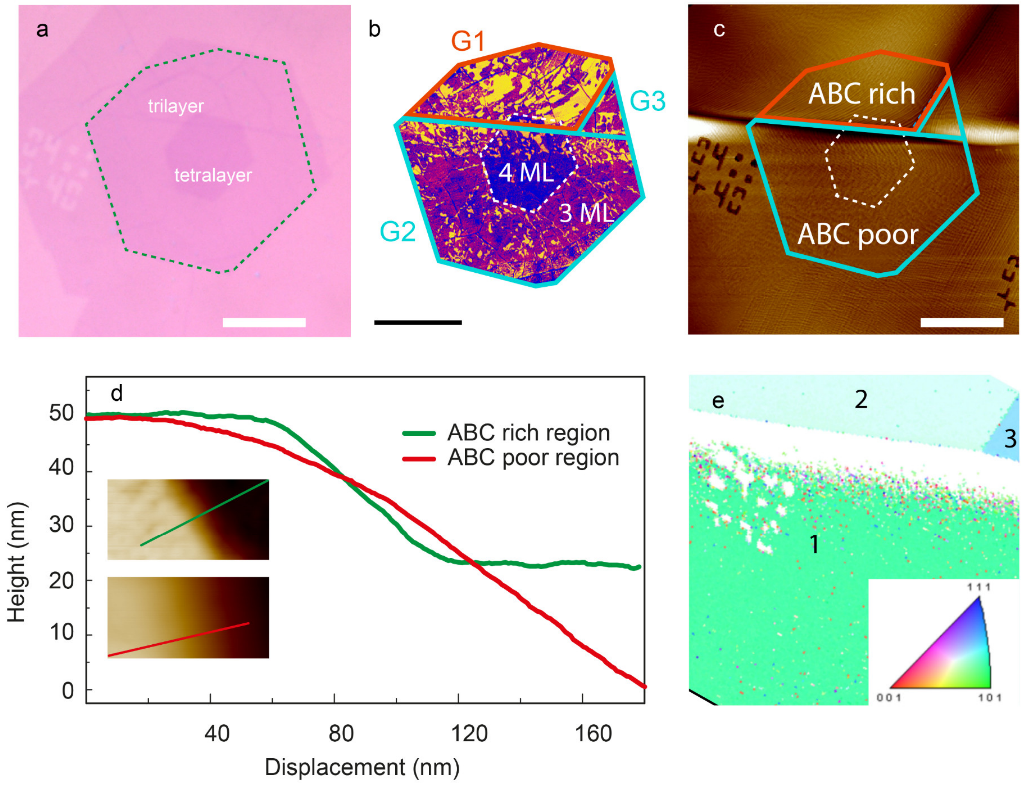

Supplementary Figure 6. Different surface crystal orientations result in different topographies and different ABC yield. **a,** Optical image and **b,** IR-SNOM map of ABA- (purple) and ABC-TLG (yellow) regions within a single flake that spans three different substrate grains (G1-G3) with different topographies. 40% (7%) ABC coverage is seen in region G1 (G2) with typical curvature ~1-3×10^{7} m^{-1} (~3-6×10^{6} m^{-1}). IR-SNOM image of a marked TLG flaked transferred onto a SiO_2 substrate, contrast scale: 2.42 V. **c**, Overlay of the TLG flake onto the corresponding AFM topography image of the growth substrate, revealing the TLG flake covers three substrate grains. **d**, Representative line profiles of the corrugations in the ABC rich and ABC-poor regions of the substrate. The inset shows the AFM topography image of the substrates. **e,** Inverse pole figure (IPF) of electron backscatter diffraction (EBSD) showing the different crystal orientation of the substrate grains. Scale bars in **a-c**: 10 µm.

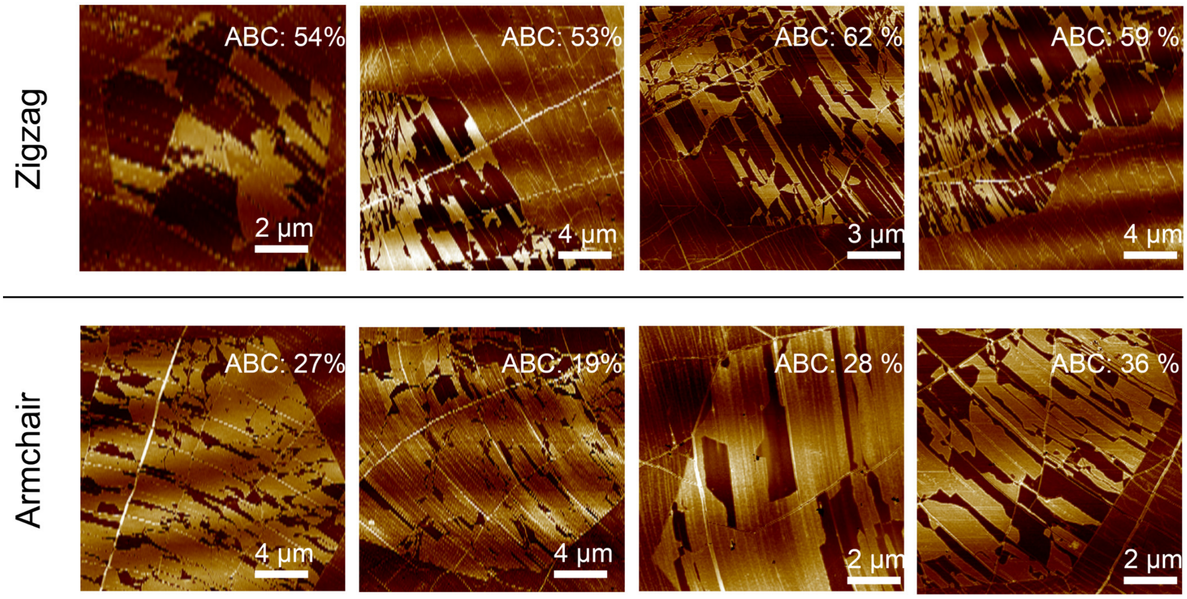

Supplementary Figure 7. IR-SNOM images showing differences in ABC yield between zigzag and armchair domain wall line directions.

Supplementary Notes

Supplementary Note 1: Density functional theory (DFT) calculations

To elucidate the growth mechanism of TLG on copper and nickel-doped copper, we model the copper substrate as a Cu (111) surface, since the majority of the surface in our experiments is composed of (111) terraces. The results are expected to be transferable to other faces. For the bare copper surface, we find that nickel prefers to occupy sites in the layer immediately below the surface rather than in the surface layer (subsurface Ni is stabilized by 0.22 eV per Ni atom relative to surface Ni). This stabilization of subsurface nickel still holds in the presence of a graphene sheet adsorbed on the surface. However, isolated carbon dimers tend to promote the movement of nickel to the surface. Therefore, and to take into account the expected statistical distribution of nickel in experiments, we model the most important carbon adsorption geometries for all anticipated substrate configurations, namely copper with subsurface nickel [Cu(Ni_{sub})], with surface nickel [Cu(Ni_{surf})], and pristine copper [Cu].

To gain insight into early-stage graphene growth, we first compare the stability of different carbon dimers on the surface[2,3]. In order to differentiate different arrangements, we label the carbon atoms (see **Supplementary Fig. 8**) according to their adsorption sites on the (111) surface as "top" (directly over a substrate atom), "fcc" (in a C site relative to the top two substrate layers in an AB configuration), and "hcp" (in an A site relative to the top two substrate layers in an AB configuration). Dimers are labeled by a pair of adsorption sites *X*-*Y* (top-fcc, top-hcp, or fcc-hcp). We compare a variety of different carbon dimer adsorption structures, including those near nickel sites on Cu(Ni_{surf}), near Ni sites on Cu(Ni_{sub}), and away from any Ni dopant (see **Supplementary Fig. 8** and **Supplementary Table 1**). Whenever carbon atoms are located over Ni atoms (e.g., **Supplementary Fig. 8e**), this is noted with Ni in parenthesis after the respective surface site. For dimers in the direct vicinity of a Ni dopant, the relative orientation is of importance. The C-C bond of a dimer can be either parallel (||) or perpendicular ($\perp$) to the axis connecting the center of the C-C bond and the Ni dopant. Suffixes "iso" and "sub" denote situations of a dimer being isolated, several atoms away from the nearest Ni dopant or in the vicinity of a subsurface dopant, respectively.

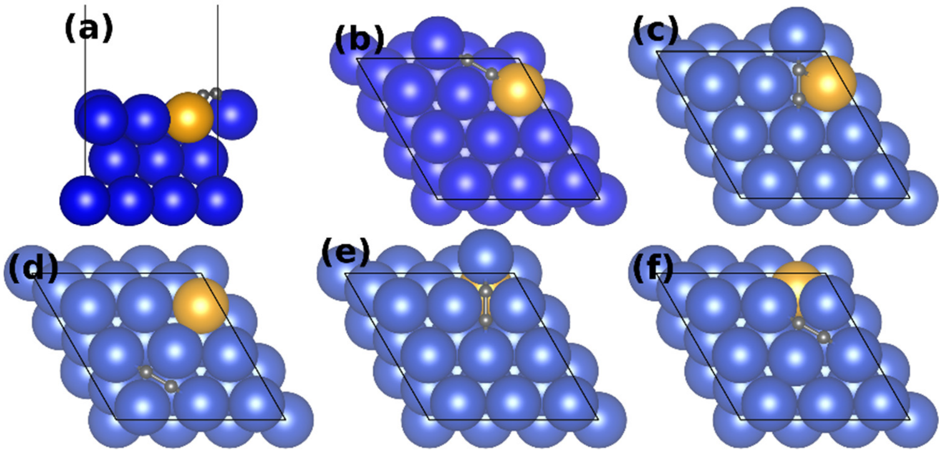

Supplementary Figure 8. Stable structures of a carbon dimers on Ni-doped Cu (111). The blue atoms represent Cu, the gold atoms Ni, and the small gray atom pairs are carbon dimers. **a**, Side- and **b**, top-view of the fcc-hcp dimer structure with C-C bond parallel to the Ni-C axis (fcc-$hcp_{||}$), **c**, fcc-$hcp_{\perp}$ with C-C bond

perpendicular to the Ni-C axis, **d**, isolated fcc-hcp$_{iso}$, **e**, fcc-hcp over subsurface Ni (fcc-hcp(Ni)), and **f**, fcc-hcp$_{sub}$ next to subsurface Ni.

We find that the top site (over Cu or Ni) is always unfavorable compared to fcc and hcp sites, in line with results[6] for an isolated carbon atom on pristine Cu (111) (not shown), i.e., stable dimer structures always adsorb on fcc and hcp sites. By avoiding top sites, carbon atoms are adsorbed closer to the surface, maximizing their electronic interactions with the surrounding substrate sites. Proximity to a Ni surface dopant is energetically most favorable, particularly with the C-C bond perpendicular to the Cu-Ni axis. These results suggest that there is a driving force for nickel dopants to assemble on the surface, although this is accompanied by a small energy penalty on the clean surface or once a graphene sheet has formed (see below). Furthermore, this demonstrates that during growth, carbon structures initially form in fcc-hcp arrangements, in line with results for small carbon clusters[4].

We proceed by comparing different adsorption geometries of graphene sheets and stacking arrangements of multiple sheets. We employed a notation in which the respective adsorption sites of carbon relative to the surface are labeled (again as top-fcc, top-hcp, or fcc-hcp), and multiple graphene sheets (and the substrate) are separated by a slash "/". Graphene on copper is known to be physiosorbed[4-6], with a preference for top-fcc and top-hcp over fcc-hcp configurations (see **Supplementary Fig. 9**). This is reproduced in our calculations with a stabilization of top-fcc and top-hcp sites by ~4 meV per atom and an averaged adsorption distance of 3.28 Å.[4,5,7] Note that additional, slightly destabilized, bridge adsorption sites were disregarded here as we seek to find energetically preferable arrangements. There is a small preference for the top-fcc over the top-hcp arrangement (~0.6 meV per atom).

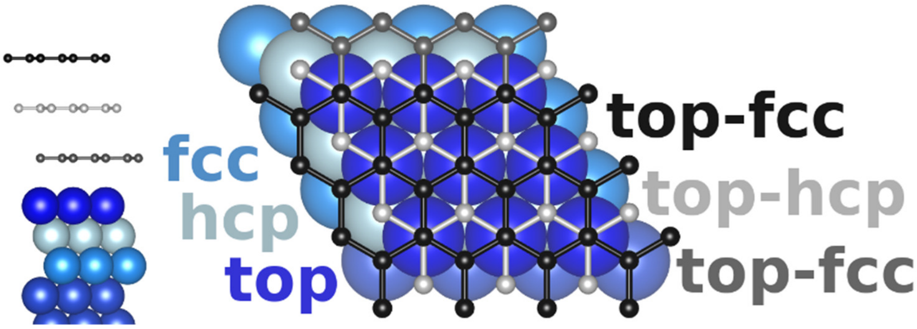

Supplementary **Figure 9.** Structure of top-fcc/top-hcp/top-fcc/Cu demonstrating the chosen naming convention for (multilayer) graphene adsorbed on copper.

Supplementary Table 1: Binding energies E_b of carbon dimers, graphene, and multilayer graphene on pristine and nickel doped Cu (111), per carbon atom. Arrows denote spontaneous relaxation to the indicated structure.

Structure	E_b (eV/atom)
**C—C Dimer**	
fcc-hcp$_{\|\|}$	0.922(7)
fcc-hcp$_{\perp}$	0.881(0)
top(Ni)-fcc	⟶ fcc-hcp$_{\|\|}$
top(Ni)-hcp	⟶ fcc-hcp$_{\|\|}$
fcc-hcp$_{iso}$	1.095(0)
fcc-hcp(Ni)	1.104(4)
top-hcp(Ni)	⟶ fcc-hcp(Ni)
fcc-hcp$_{sub}$	1.092(6)
**Bilayger Graphene**	
top-fcc/Cu	-0.113(4)
top-hcp/Cu	-0.112(8)
fcc-hcp/Cu	-0.108(8)
top-fcc/Cu(Ni$_{surf}$)	-0.120(3)
fcc-hcp/Cu(Ni$_{surf}$)	-0.110(7)
top-fcc/Cu(Ni$_{sub}$)	-0.118(6)
fcc-hcp/Cu(Ni$_{sub}$)	-0.108(9)
**Trilayer Graphene**	
AB top-fcc/fcc-hcp/Cu	-0.095(4)
AB top-fcc/top-hcp/Cu	-0.097(9)
AB fcc-hcp/top-fcc/Cu	-0.098(3)
AA fcc-hcp/fcc-hcp/Cu	-0.088(7)
ABC top-fcc/top-hcp/hcp-fcc/Cu	-0.091(3)
ABC top-fcc/fcc-hcp/top-hcp/Cu	-0.094(7)
ABC fcc-hcp/top-fcc/top-hcp/Cu	-0.094(5)
ABC top-hcp/fcc-hcp/top-fcc/Cu	-0.095(4)
ABC fcc-hcp/top-hcp/top-fcc/Cu	-0.095(3)
ABA top-fcc/top-hcp/top-fcc/Cu	-0.095(3)
ABA top-fcc/fcc-hcp/top-fcc/Cu	-0.095(5)
ABA top-hcp/top-fcc/top-hcp/Cu	-0.095(3)
ABA top-hcp/fcc-hcp/top-hcp/Cu	-0.094(5)
ABC top-fcc/top-hcp/hcp-fcc/Cu(Ni$_{surf}$)	-0.091(9)
ABC top-fcc/fcc-hcp/top-hcp/Cu(Ni$_{surf}$)	-0.095(2)
ABA top-fcc/top-hcp/top-fcc/Cu(Ni$_{surf}$)	-0.095(5)
ABA top-fcc/fcc-hcp/top-fcc/Cu(Ni$_{surf}$)	-0.095(6)
ABC top-fcc/top-hcp/hcp-fcc/Cu(Ni$_{sub}$)	-0.091(3)
ABA top-fcc/top-hcp/top-fcc/Cu(Ni$_{sub}$)	-0.094(8)

We, however, think that DFT energy *differences* smaller than 1 meV per atom should be interpreted conservatively and that systems with such small energy differences may be viewed as isoenergetic. We indicate this by reporting the sub-meV per atom digit only in parentheses. However, for graphene sheets on a substrate as discussed here, many similar arrangements exist and often a clear trend can be identified upon comparing different arrangements. This may help to

identify absolute thermodynamic minima, whereas under realistic conditions such small energy differences may lead to a statistical average over all nearly-isoenergetic arrangements. For example, for the case of top-fcc vs. top-hcp arrangements, the former is always slightly more stable and we, therefore, discuss the results accordingly, noting, however, that the quantitative energy differences may be beyond the accuracy of our method.

When including the effect of Ni doping, the preference for top-fcc over fcc-hcp adsorbed graphene increases to about 10 meV per atom. The influence of the Ni position (surface vs. subsurface nickel) on these energy differences is negligible. Overall, extended graphene sheets prefer top-fcc and top-hcp sites, which differs from the carbon dimer. This difference can be explained by the altered interactions with the surface, from dangling bonds bound strongly to the surface (for a dimer) to a weakly van der Waals bound graphene sheet with a larger adsorption distance. Thus, while very small carbon flakes on Cu seem to prefer an fcc-hcp structure during bottom-up growth, periodic graphene sheets prefer a top-fcc or top-hcp arrangement. This suggests that upon growth, graphene flakes reach a critical size at which fcc-hcp adsorption becomes less favorable than top-fcc or top-hcp adsorption, and the flakes then prefer to slide over the surface to occupy the more stable adsorption state.

To estimate the likeliness of such a slide, we computed the path between a periodic fcc-hcp absorbed graphene sheet and a top-fcc sheet. We find that such a transition is for periodic sheets barrier-less. This suggests a dynamical behavior during the growth of graphene flakes on our Ni-Cu alloy substrate, with sufficiently large graphene patches shifting from the initial thermodynamically preferred fcc-hcp adsorption sites to the final thermodynamically preferred top-fcc or top-hcp adsorption sites. Although this transition in nonperiodic (finite-size) flakes may involve an energy barrier, barriers will likely decrease with increasing flake size. The possibility for such a dynamical behavior of graphene flakes has been observed previously on graphite[8,9].

During back-diffusion growth, new graphene sheets grow at the alloy substrate surface[10], i.e., once the first layer is grown, it is lifted from the substrate by a new layer and this process is repeated with each new layer lifting the upper layers. The above predicted shifting of graphene relative to the substrate surface during growth may impact the kinetic accessibility of thermodynamically stable TLG states, and therefore warrants further study. However, we find that greater insight is provided at present by energetic considerations. We therefore proceed by analyzing different layer stackings from a total energy perspective.

Neglecting different orientations, sheets in multilayer graphene can be translated relative to each other into stable positions, leading to three distinguishable stacking positions (A, B, and C) that we denote here with respect to the Cu(Ni) substrate. There is a variety of different possibilities for stacking these layers. As mentioned above, it is known that any AA-type stacking is unfavorable and that interlayer interactions are stabilized in an AB arrangement, which can be rationalized by tight-binding models[11,12]. In natural graphite this results in predominantly ABA stacking, whereas ABC stacking is observed in smaller quantities of about 5-15%. The content of the latter can usually be reduced by heating, although it is reported that ABC regions are stable up to elevated temperatures of 1,000 – 1,200 °C[13]. In any case, the energy surface associated with shifting the different layers (e.g., from ABC to ABA) is extremely flat with small energy differences. Consequently, the energies of both stackings are nearly isoenergetic. For example, at the level of the local-density approximation, the energy difference between ABA and ABC graphite is 0.1-0.2 meV per atom[14,15]. This is in line with our calculations at the PBE+TS level with an energy difference of 0.2 meV per atom in the bulk and 0.07 meV per atom for free-standing TLG. We also employed more recent approaches, namely PBE+TSMBD[16] and HSE+TSMBD[17].

In agreement with earlier results, these calculations predict, in contrast to graphite, that ABC is preferred over ABA by a very small energy difference of ~0.2 meV per atom. As noted above, such small energy differences are at the edge of DFT accuracy and we conclude that ABA and ABC are nearly isoenergetic. Based on the noted observations concerning graphite, we focus on the more computationally efficient and sufficiently accurate PBE+TS method which yields a very slight energy preference for ABA. Most importantly, however, we conclude that the coexistence of ABA and ABC stackings in many cases suggests that the thermodynamic energy differences are small enough to invite the expectation that other factors may control the resulting structures.

With this in mind, we can directly compute the energies of different graphene multilayer structures on copper (see **Supplementary Table 1**). As for single-layer graphene, we find that structures with a top-fcc or top-hcp adsorbed surface layer are favored over fcc-hcp adsorbed layers due to the stronger interaction of the surface layer with the substrate. This is observed on pristine copper and both of our doped structures, for single-, bilayer-, and trilayer graphene. In all cases, the preference for the top-fcc and top-hcp arrangements over fcc-hcp is ~3-5 meV per atom. The energy differences between top-fcc and top-hcp surface layers are again an order of magnitude smaller than this, with a preference for top-fcc of ~0.4-0.8 meV per atom.

Overall, we can rank the various TLG orderings on a Cu(Ni) substrate in the following way. First, any states with AA stackings are the least stable. Second, for the remaining ABA and ABC stackings, the bottom-most layer in direct contact with the substrate dominates the stability. In line with results for single-layer graphene, hcp-fcc adsorption is destabilized compared to top-fcc and top-hcp adsorption sites. Last, although top-fcc and top-hcp are close in energy, there is a small preference for top-fcc sites. With these observations, we conclude that there are at least two possible preferred ABA [top-fcc/fcc-hcp/top-fcc and top-fcc/top-hcp/top-fcc (top-hcp/fcc-hcp/top-hcp and top-hcp/top-fcc/top-hcp)] configurations and the same number of preferred ABC configurations [top-hcp/fcc-hcp/top-fcc and fcc-hcp/top-hcp/top-fcc (top-fcc/fcc-hcp/top-hcp and fcc-hcp/top-fcc/top-hcp)]. The relative energies and trends are consistent for the different substrates, independent of the presence and position of the nickel dopant. For pristine copper, we computed all possibilities and find that the fcc-hcp orientation in the second layer is most stable.

These results suggest that both ABA- and ABC-TLG stackings on flat Cu(Ni) are thermodynamically accessible (and nearly isoenergetic) within the accuracy of our calculations. Furthermore, the near barrier-less shifting of graphene sheets on (nickel doped) copper (and the fact that small graphene seeds are likely to change position during growth) means that both ABA- and ABC-TLG can in principle be realized on our Ni-Cu gradient alloy substrates. However, we cannot make any predictions regarding the relative occurrences of these two stackings or the effects of non-idealized, non-flat substrates. We therefore consider the possibility that corrugations of the substrate are a cause of the selection of different stackings in different regions.

Supplementary Note 2: First principles-informed continuum model for weakly bound layered materials on non-flat surfaces

The total energy of an elastic multilayer system is a function of the spatially varying curvature-induced in-plane strain $\varepsilon_\kappa(x)$ and the number and type of interlayer dislocations. We decompose this energy into contributions from curvature-induced in-plane disregistry strain $\varepsilon_\kappa(x)$, interlayer dislocation line energies, and stacking phase bulk energies,

$$E_{\text{tot}} = E_{\text{disreg}}\{\varepsilon_\kappa(x)\} + E_{\text{disloc}} + E_{\text{phase}}.$$

For trilayer graphene on corrugated Cu (111), the substrate topography is assumed to vary only along x such that the curvature-induced in-plane disregistry strain energy over a region of size L_{Cu} (per unit length y) is

$$E_{\mathrm{disreg}} = \frac{E^{\mathrm{2D}}}{2}\sum_{i=1}^{3}\int_{0}^{L_{\mathrm{Cu}}}\left[\varepsilon_i + \varepsilon_{\kappa,i}(x)\right]^2 dx,$$

where $\varepsilon_i = b_i N_i - \varepsilon_{0,i}$ is the elastic strain and $\varepsilon_{\kappa,i}(x) = d_i/[\kappa^{-1}(x) + \sum_{j=i+1}^{3} d_j]$ is the geometric strain. $E^{\mathrm{2D}} = 340\ \mathrm{N\ m^{-1}}$ [18] is the 2D Young's modulus of graphene, i is the interlayer index ($i =$ 1, 2, and 3 correspond to C_1-C_2, C_2-C_3, and C_3-Cu, respectively; see **Fig. 3b**), b_i is the dislocation Burgers vector magnitude along x (edge component), N_i is the integer dislocation density between $x = 0$ and $x = L_{\mathrm{Cu}}$, $\varepsilon_{0,i}$ is the in-plane misfit strain between flat layers in i, and the interlayer spacing is $d_i = 0.335$ nm [19] is the interlayer separation. We separate geometric strains from elastic strains in E_{disreg} to emphasize the physical competition between these two effects. Geometric strain $\varepsilon_{\kappa,i}$ is that introduced *via* curvature under the condition of epitaxial registry between layers (as illustrated in **Fig. 3b**), and elastic strain ε_i is that introduced by interlayer dislocations. Without dislocations, the entire geometric strain is stored as elastic energy within the system. With a particular amount of dislocations, the introduced elastic strain exactly offsets the geometric strain, and the strain energy goes to zero. With this form of E_{disreg}, the strain reference state is that which a given graphene layer rests strain-free on either the substrate (*i*=3) or the graphene layer below it (*i*=1 or 2). This form also invokes the assumption that dislocations relieve strain uniformly over each local peak or valley. This energy is minimized when $\varepsilon_i = \varepsilon_{\kappa,i}(x)$, which corresponds to a geometrically preferred dislocation density that perfectly cancels $\varepsilon_{\kappa,i}(x)$. Since $\varepsilon_{0,1} = \varepsilon_{0,2} = 0$ here, if we neglect $i = 3$ (the substrate), then only the substrate topography, contained within $\varepsilon_{\kappa,i}(x)$, must be specified. We describe the curvature as either localized $\kappa(x) = \kappa_0 \exp(-x^2/2\sigma^2)$ as described in the main text or periodic $\kappa(x) = -A_{\mathrm{Cu}}(2\pi/L_{\mathrm{Cu}})^2 \sin(2\pi x/L_{\mathrm{Cu}})$ as described below. Bending energy differences between states can be neglected (the bending modulus of graphene is extremely small).

Interlayer dislocations relieve curvature-induced interlayer strain but also introduce dislocation line energy (i.e., domain wall energy). This implies that the geometrically preferred dislocation density that minimizes E_{disreg} is in general larger than the dislocation density that minimizes E_{tot}. A balance between the competing contributions from E_{disreg} and E_{disloc} must be found. The total dislocation line energy (per unit length y, adapted from Dai *et al.*[20]) is expressed as

$$E_{\mathrm{disloc}} = \sum_{i=1}^{3} L_{\mathrm{Cu}} N_i \left[\frac{b_i}{a}\left(E_{\mathrm{edge}}\sin^2\theta_i + E_{\mathrm{screw}}\cos^2\theta_i\right) + \frac{2b_i^2}{L_{\mathrm{Cu}}}\left(\frac{E^{\mathrm{2D}}}{4} + \frac{B}{d_i^2}\right)\right],$$

where $a = 0.14$ nm is the nearest neighbor distance in graphene, $E_{\mathrm{edge}} = 0.318 \times 10^{-10}\ \mathrm{J\ m^{-1}}$ and $E_{\mathrm{screw}} = 1.091 \times 10^{-10}\ \mathrm{J\ m^{-1}}$ are the core energy coefficients, θ_i is the angle between the dislocation line and Burgers vector directions, and $B = 22.08 \times 10^{-20}$ J is the bending modulus of graphene. The first term in the square brackets is the core energy and the second term is the contribution from long-range interactions between interlayer dislocations.

E_{phase} is the total bulk energy of the periodic ABA/ABC system considered here, and is given by $E_{\mathrm{phase}} = E_i L_{\mathrm{Cu}} \sum_{j=1}^{3} d_j$ for single phase states (E_i is the bulk energy of ABA or ABC per

unit volume) and by $E_{\text{phase}} = \frac{1}{2}(E_{\text{ABA}} + E_{\text{ABC}})L_{\text{Cu}}\sum_{j=1}^{3} d_j$ for two phase states. Since $E_{\text{ABA}} < E_{\text{ABC}}$ (though by a very small amount), E_{phase} favors dislocation-free ABA states, followed by dislocation-containing ABA/ABC states, and finally by dislocation free ABC states.

The dislocation line direction (orientation of the domain walls) is fixed by the orientation of the TLG lattice relative to that of the substrate corrugations. Given this line direction and a specific substrate topography, we can straightforwardly minimize E_{tot} with respect to the dislocation spacing for each of the ten states outlined in the main text. These states were chosen based on the crystallographically allowed interlayer dislocation types in stacked graphene.

Such dislocations may be partials ($\mathbf{b} = a\langle 1\bar{1}00\rangle/\sqrt{3}$), which require a change in relative stacking across each partial-dislocation line, or perfect dislocations ($\mathbf{b} = a\langle 11\bar{2}0\rangle/\sqrt{3}$), which involve no change in relative stacking[20,21]. An initially AB bilayer, for example, bisected by an interlayer partial-dislocation becomes AB on one side of the dislocation line and AC on the other. Similarly, a single interlayer partial-dislocation in a trilayer is an ABA/ABC-type boundary, while a vertically stacked pair of interlayer partials is an ABA/ACA-type or ABC/ACB-type boundary (**Fig. 3c**).

Configurations with boundaries containing less edge character (screw-dominant) than those considered here may also exist, but these will have greater total energy than their counterparts with equal total $|\mathbf{b}|$ in **Fig. 3c**. This is because screw-dominant boundaries introduce comparable dislocation energy to their edge-dominant counterparts but relieve less curvature energy (the screw component relieves only shear strain while 1D curvature strain is axial).

The unexpected stabilization of states containing the ABC phase (**Fig. 3e**) results from the difference in geometrically allowed partial-dislocation content between ABA and ABC stacking variants (E_{disreg}) and the corresponding difference in dislocation energies (E_{disloc}). As shown in **Fig. 3d**, ABC/ACB configurations ($\mathbf{z}$-$\mathcal{R}_2^2\mathcal{R}$) accommodate 1D curvature strain more efficiently than ABA/ACA ($\mathbf{z}$-$\mathcal{B}_2^1\mathcal{B}$), due to the greater edge character of $\mathbf{z}$-$\mathcal{R}_2^2\mathcal{R}$ interfaces. The ABC phase is therefore preferred over a range of intermediate curvatures despite its larger bulk energy. This difference disappears only at the armchair line direction ($\mathbf{a}$-$\mathcal{B}_{\sqrt{3}}^{\sqrt{3}}\mathcal{B}$ and $\mathbf{a}$-$\mathcal{R}_{\sqrt{3}}^{\sqrt{3}}\mathcal{R}$ relieve the same amount of curvature strain, see **Supplementary Fig. 10**), such that the energetic advantage of the ABC phase persists at all intermediate line directions. The equilibrium $\mathbf{z}$-$\mathcal{R}_2^2\mathcal{R}$ region terminates when the curvature is large enough to accommodate full dislocations and $\mathbf{z}$-$\mathcal{B}_4^4\mathcal{B}$ becomes the equilibrium state.

The existence of a $\mathbf{z}$-$\mathcal{R}_2^2\mathcal{R}$ equilibrium region requires a bulk energy difference $\Delta E = E_{\text{ABC}} - E_{\text{ABA}} \lesssim 0.1$ meV per atom; above this value the increase in E_{phase} associated with ABC domains outweighs the decrease in E_{disreg} and E_{disloc}. This ΔE is consistent with our *ab initio* calculations, which detect no difference within an estimated accuracy of ~1 meV per atom. As ΔE is decreased below 0.1 meV per atom, the $\mathbf{z}$-$\mathcal{R}_2^2\mathcal{R}$ equilibrium regions quickly converge to those shown in **Fig. 3e** for all $\Delta E \lesssim 0.02$ meV per atom (which was chosen smaller than the approximate DFT difference of 0.06 meV per atom to ensure we do not over estimate this effect).

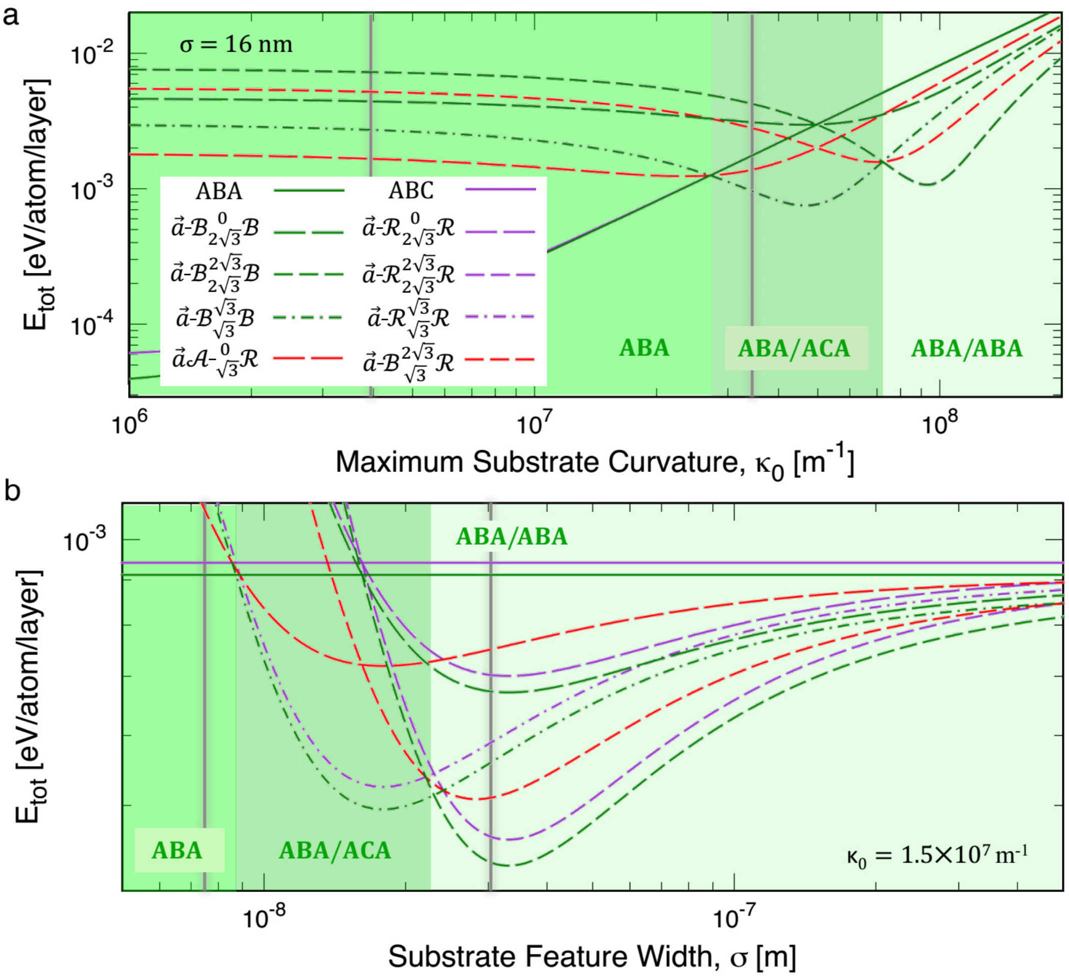

Supplementary Figure 10. Computed energies E_{tot} of the states in **Fig. 3c** with armchair line direction vs. substrate corrugation **a**, maximum curvature κ_0 and **b**, feature width σ for $\kappa(x) = z''(x) = \kappa_0 \exp(-x^2/2\sigma^2)$. The zigzag direction is discussed in the manuscript. Colored regions correspond to different equilibrium states, and brackets enclose approximate experimental values. The purple lines (ABC) in **a** are indistinguishable from the green lines (ABA) except near the lower left corner of the plot. The bulk preference for ABA of 0.02 meV per atom is present in all cases but is not visible over most of the energy scale shown in **a**. This difference is however visible in **b**.

A more general geometric parameterization than $\kappa(x) = z''(x) = \kappa_0 \exp(-x^2/2\sigma^2)$ is a sinusoidal topography, $z(x) = A_{\text{Cu}} \sin(2\pi x/L_{\text{Cu}})$ where $\kappa_{\max} = A_{\text{Cu}}(2\pi/L_{\text{Cu}})^2$. We computed the energies of the various configurations with this parameterization, and as shown in **Supplementary Figs. 11** and **12**, the results are very similar to those obtained with the Gaussian $\kappa(x)$ parameterization. This demonstrates that the relative energies of the configurations are not highly sensitive to the form of the curvature; its magnitude is the primary factor. We note, however, that for either parameterization, two variables (κ_0 and σ or A_{Cu} and L_{Cu}) are required to fully characterize systems with spatially varying $\kappa(x)$ due to the nonlinearity of E_{tot}.

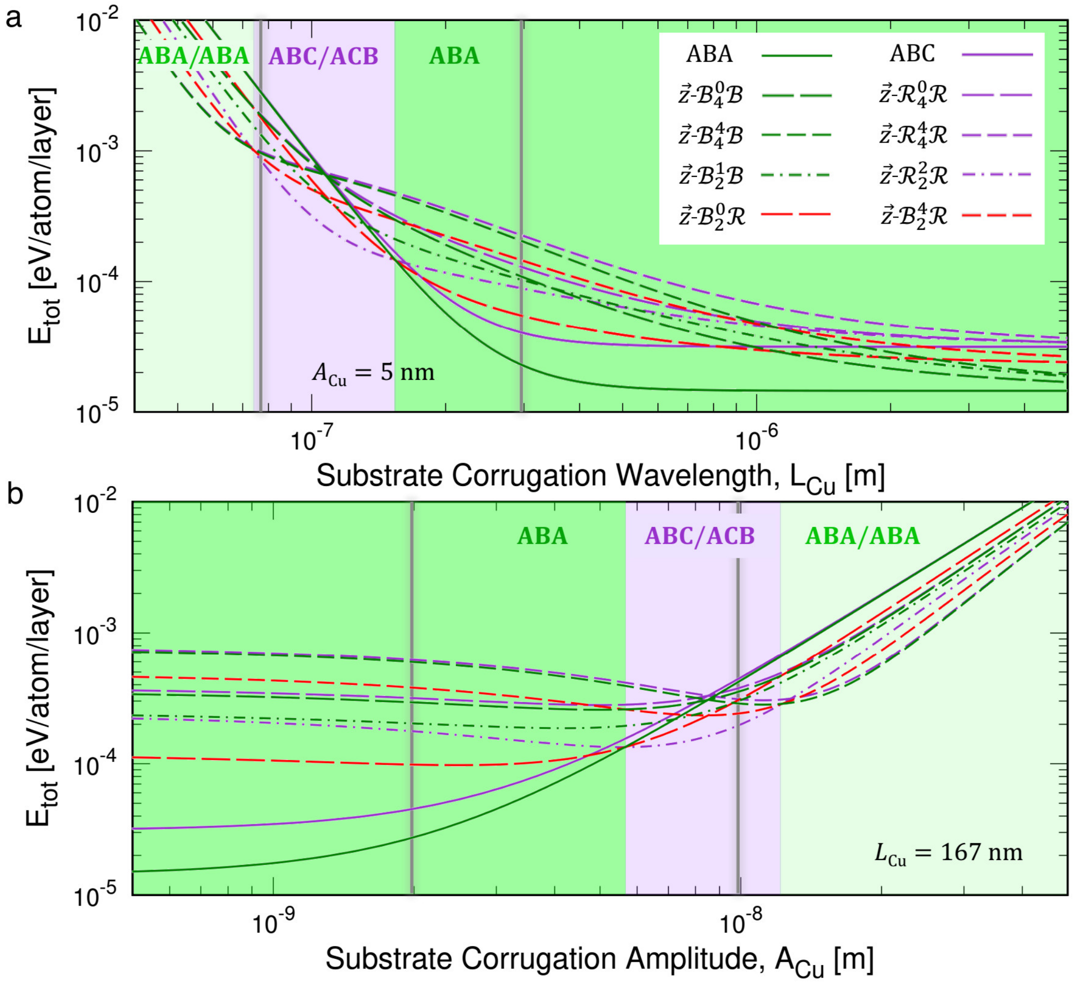

Supplementary Figure 11. Computed energies E_{tot} of the states in **Fig. 3c** with zigzag line direction vs. substrate **a**, corrugation spacing L_{Cu} and **b**, amplitude A_{Cu} for $z(x) = A_{\mathrm{Cu}} \sin(2\pi x/L_{\mathrm{Cu}})$. Colored regions correspond to different equilibrium states, and brackets enclose approximate experimental values.

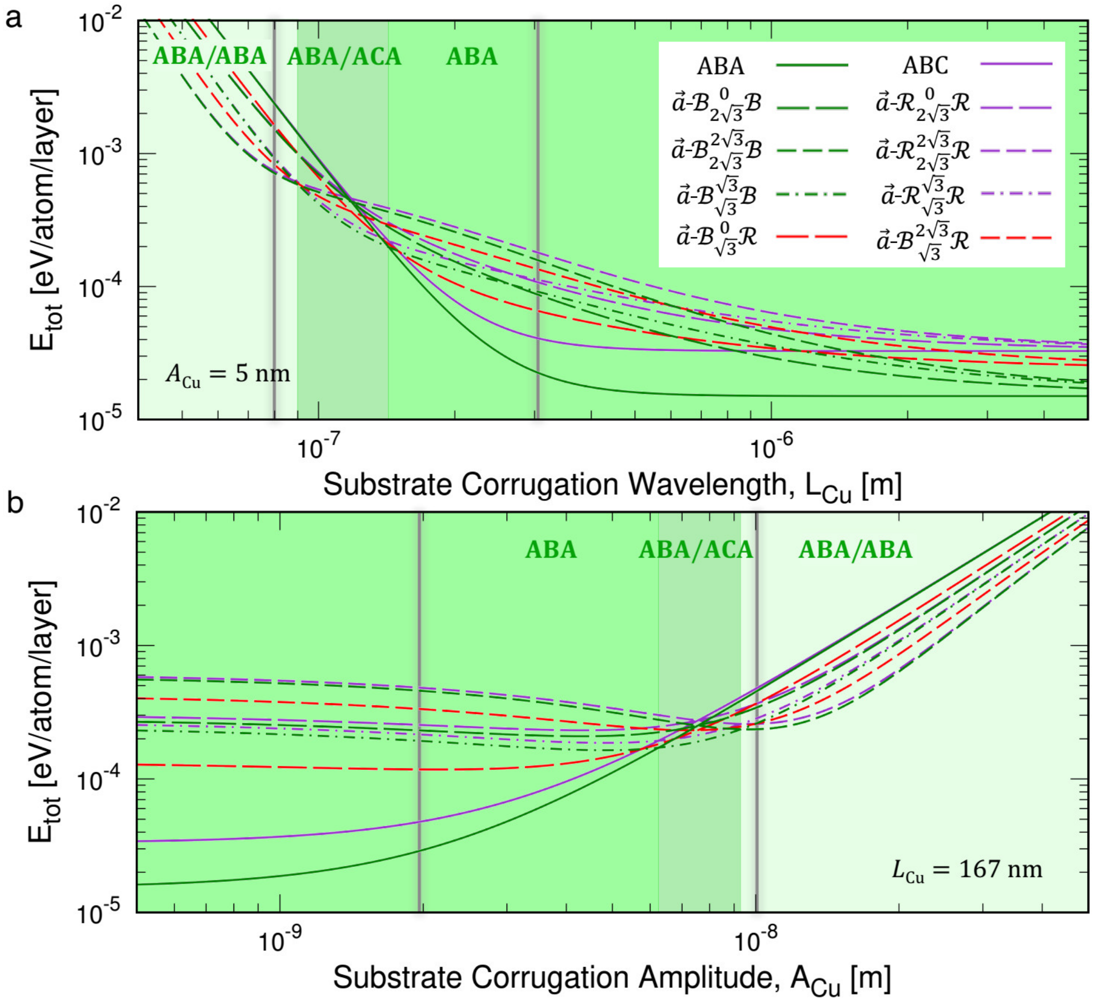

Supplementary Figure 12. Computed energies E_{tot} of the states in **Fig. 3c** with armchair line direction vs. substrate **a**, corrugation spacing L_{Cu} and **b**, amplitude A_{Cu} for $z(x) = A_{\text{Cu}} \sin(2\pi x/L_{\text{Cu}})$. Colored regions correspond to different equilibrium states, and brackets enclose approximate experimental values.

Supplementary Fig. 13 shows the separate contributions from E_{disreg}, E_{disloc}, and E_{phase} for ABA, ABC, $\mathbf{z}$-$\mathcal{B}_4^4\mathcal{B}$, $\mathbf{z}$-$\mathcal{R}_2^2\mathcal{R}$, and $\mathbf{z}$-$\mathcal{B}_2^1\mathcal{B}$ states. E_{disreg} plays a role in stabilizing the ABC phase, as does E_{disloc} due to the difference between edge and screw core energies ($E_{\text{screw}} \approx 3.4E_{\text{edge}}$). $\mathbf{z}$-$\mathcal{R}_2^2\mathcal{R}$ is fully edge while $\mathbf{z}$-$\mathcal{B}_2^1\mathcal{B}$ has a large screw component.

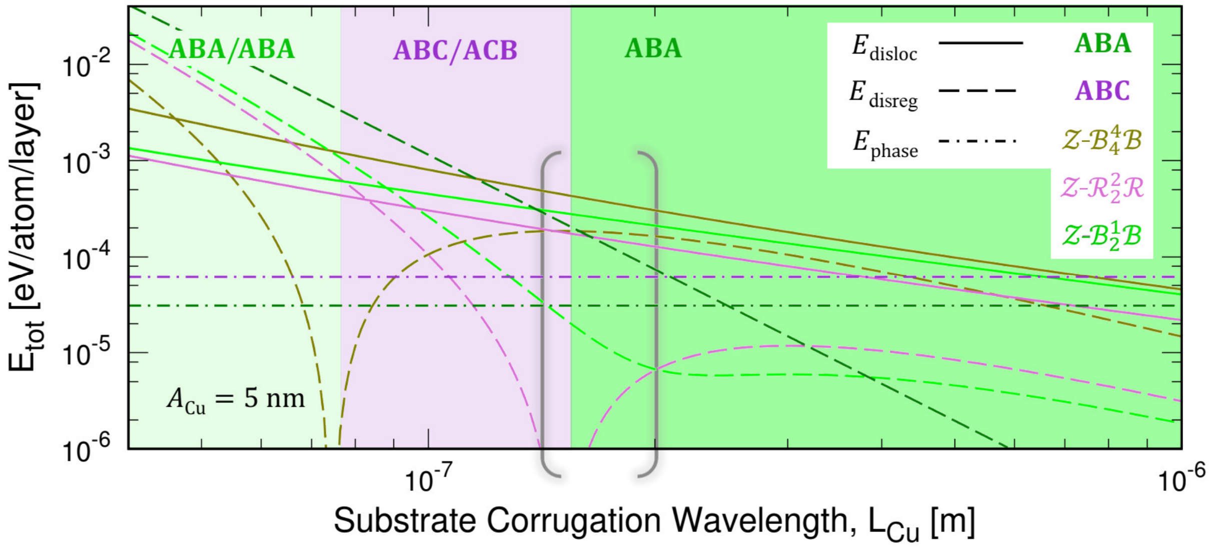

Supplementary Figure 13. Computed contributions to E_{tot} of selected states vs. L_{Cu} for a sinusoidal substrate approximation. Colored regions correspond to different equilibrium states, and brackets enclose approximate experimental values.

The results shown in **Figs. 3** and **Supplementary Figs. 10-13** correspond to $N_i = 2/L_{\mathrm{Cu}}$ (one dislocation per $L_{\mathrm{Cu}}/2$). **Supplementary Fig. 14** shows energies obtained after further minimizing E_{tot} with respect to N_i. The results are not significantly different from those shown in **Fig. 3e** and confirm that the $N_i = 2/L_{\mathrm{Cu}}$ approximation is valid over most of the range of interest. Larger dislocation densities are only preferred at very high curvatures. We also note that the largest value of L_{Cu} for which the region of **z**-$\mathcal{R}_2^2\mathcal{R}$ stability persists (at $A_{\mathrm{Cu}} \approx 25$ nm) is ~400 nm. This indicates that internal **z**-$\mathcal{R}_2^2\mathcal{R}$ interfaces in a fully ABC domain cannot be completely eliminated.

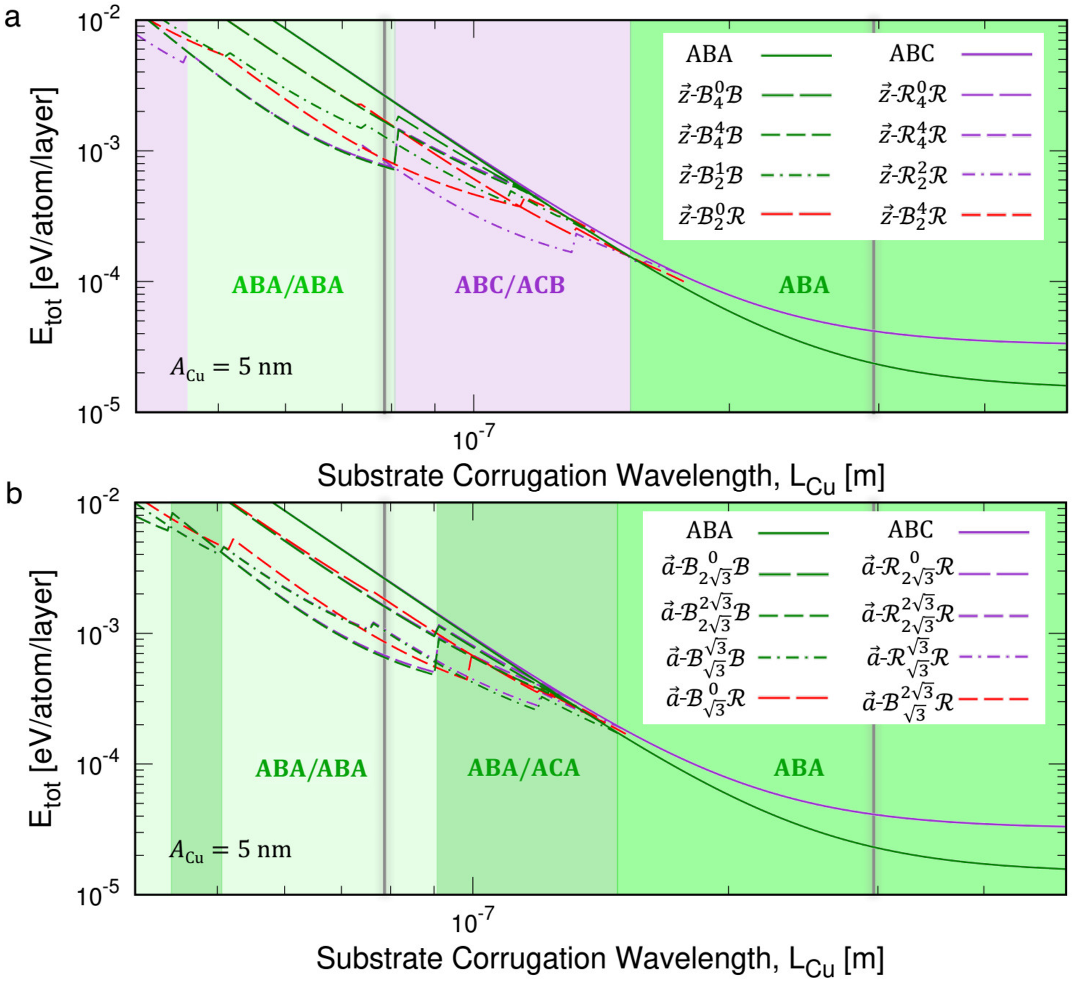

Supplementary Figure 14. **a**, and **b**, Computed semi-discrete energies E_{tot} of the states in **Fig. 3c** vs. L_{Cu} for a sinusoidal substrate approximation. Colored regions correspond to different equilibrium states, and brackets enclose approximate experimental values.

Neighboring domain walls in the symmetric substrate step edge geometry described in the main text have equal and opposite Burgers vector content, and therefore fully annihilate upon contact, leaving perfect ABA or ABC. For example, adjacent $\mathbf{z}\text{-}\mathcal{R}_2^2\mathcal{R}$ interfaces with opposite curvature produce a reaction such as $\mathbf{b}_1 + \mathbf{b}_2 = a[1\bar{1}00]/\sqrt{3} + a[\bar{1}100]/\sqrt{3} = 0$ within each interlayer (see **Supplementary Fig. 15**). Given the relatively regular, periodic growth substrate topography of our system (**Fig. 3**), many such annihilation events should occur upon transfer to SiO_2, generating significantly larger ABA and ABC domains, as observed in **Figs. 4c** and **4e**. Complete coarsening is likely suppressed by, e.g., domain wall reactions that do not result in full annihilation and pinning sites formed by dislocation junctions or substrate irregularities.

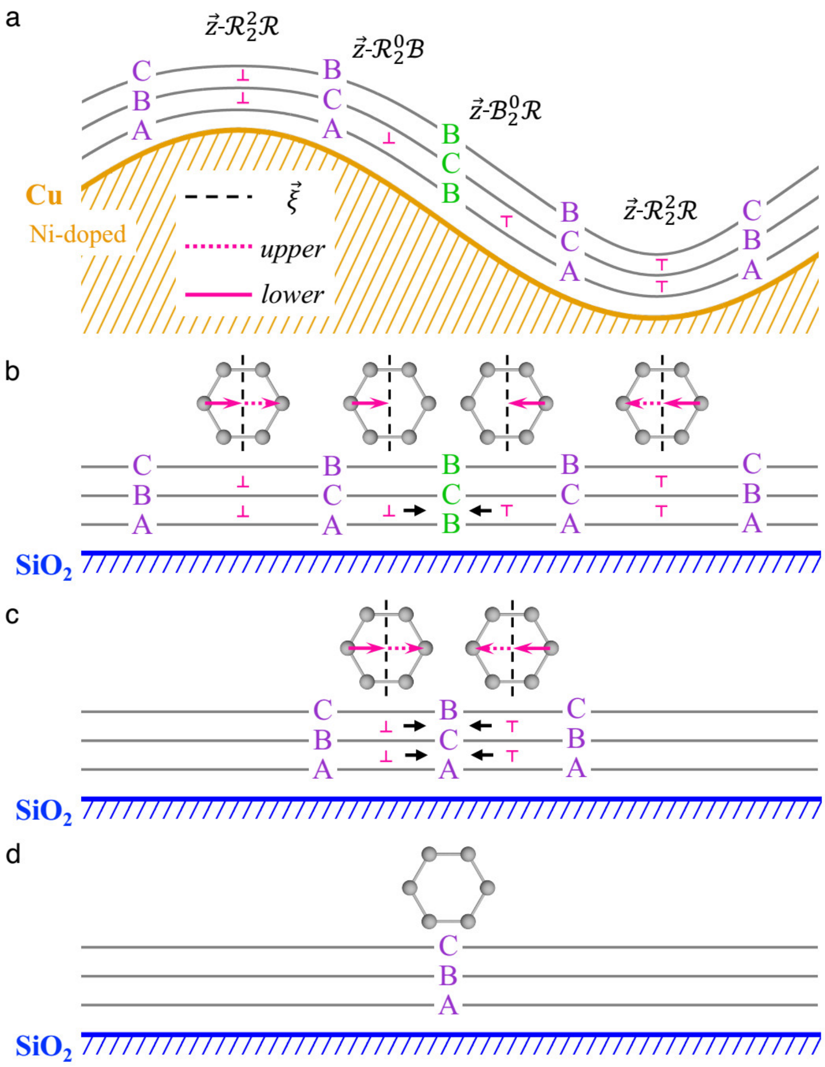

Supplementary Figure 15. Interlayer dislocation annihilation driven by transfer from the corrugated nickel-doped copper substrate to flat SiO_2. **a**, Schematic of an example post-growth morphology with four domain walls. **b-d**, Sequence of domain wall motion and annihilation events on flat SiO_2 leading to perfect ABC. The initial BCB domain is smaller than the critical size for spontaneous growth.

The small implied energetic preference for ABA may lead on average to more ABA than ABC growth after transfer, but initial domain wall morphologies that *locally* favor ABC growth are also probable. For example, the local energy of a sufficiently small ABA domain surrounded by ABC is higher than that of a fully ABC region, due to the domain wall energy associated with the ABA domain. Such a morphology will transform to a fully ABC state as long as the initial ABA domain is smaller than the "critical nucleus" size for ABA growth.

The initial morphology of a TLG flake can strongly influence its final morphology after transfer to a flat substrate. The domain wall reactions induced upon transferring a fully ABA/ACA ($\mathbf{z}$-$\mathcal{B}_2^1\mathcal{B}$) flake to a flat substrate will in general lead to the formation of a mixed ABA/ABC flake (**Supplementary Fig. 16**). A 50% probability exists for reacting domain walls to leave behind a perfect screw dislocation rather than full annihilation. Such screw dislocations spontaneously

dissociate into a pair of partials with a strip of ABC in between. However, a fully ABC/ACB (**z**-$\mathcal{R}_2^2\mathcal{R}$) flake transferred to a flat substrate will generally remain ABC/ACB. Many domain walls will fully annihilate upon contact, leading to domain growth without the introduction of ABA domains (**Supplementary Fig. 17**). Finally, a mixed ABA/ABC flake transferred to a flat substrate will in general undergo an increase in the average ABA and ABC domain sizes (**Supplementary Fig. 18**).

The coincidence of wrinkles and ABA-ABC domain walls is consistent with domain wall formation at protruding surface features (substrate corrugation extrema) and subsequent localization of mechanically-induced wrinkles along these pre-templated features during cooling and transfer. The domain walls also naturally generate a wrinkle-like out-of-plane displacement, further amplifying this pre-templating of wrinkles along domain walls. Interlayer partials, for example, in free-standing bilayer graphene locally protrude ~1-2 nm out-of-plane[7], implying that domain walls at corrugations with negative curvatures (peaks) will act as pre-nucleated wrinkles that are likely to grow if any compression is induced upon transfer.

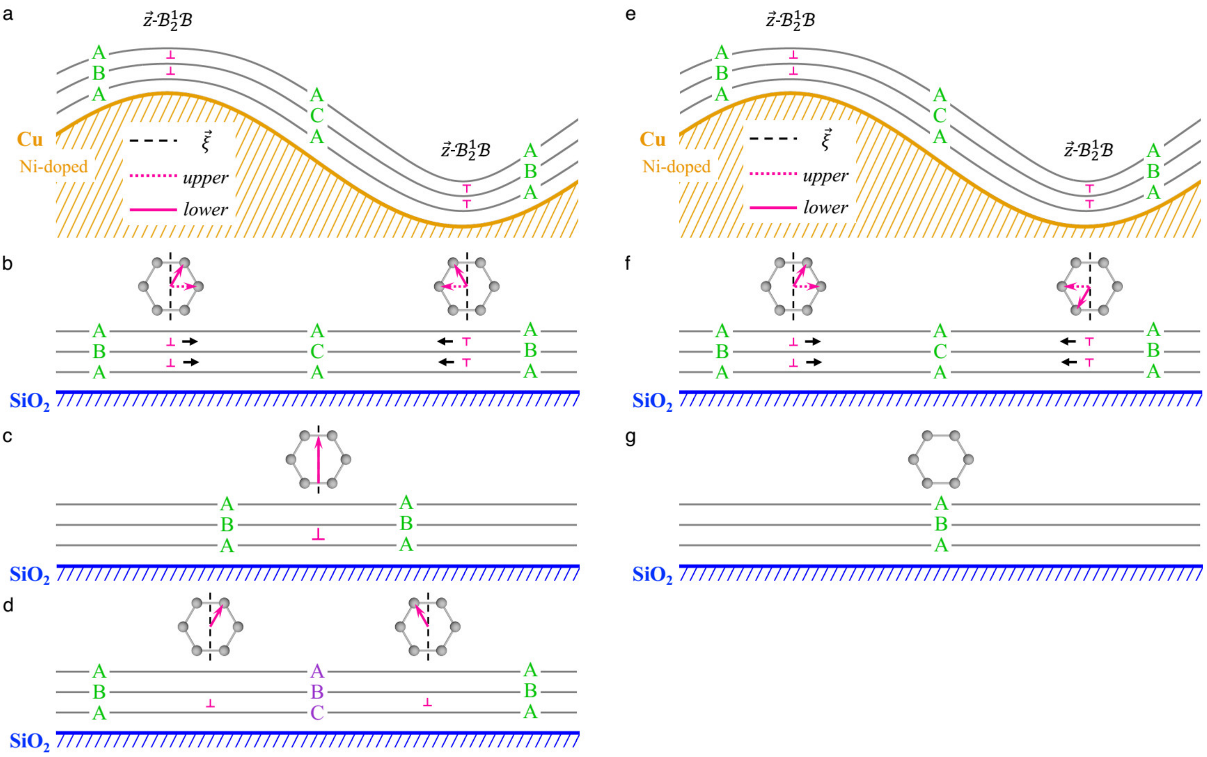

Supplementary Figure 16. Creation of ABC domains in ABA/ACA flakes transferred from the corrugated nickel-doped copper substrate to flat SiO_2. **a** and **e**, Schematics of example post-growth ABA/ACA morphologies with two domain walls. **b-d**, Sequence of domain wall motion, reaction, and dissociation on flat SiO_2 leading to a mixed ABA/ABC flake. **f-g**, Domain wall motion and reaction on flat SiO_2 leading to ABA domain growth.

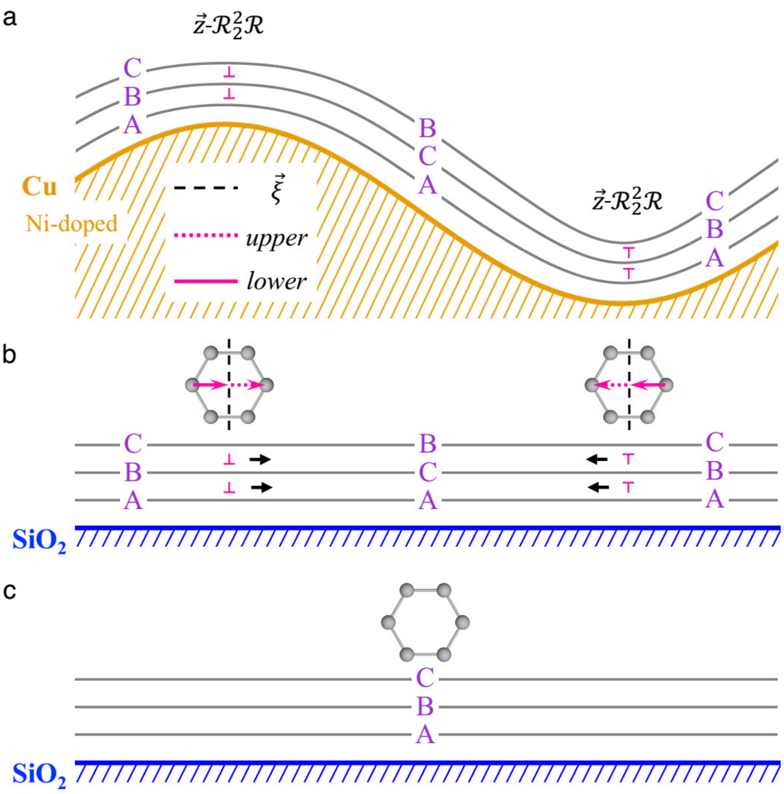

Supplementary Figure 17. Growth of ABC domains in ABC/ACB flakes transferred from the corrugated nickel-doped copper substrate to flat SiO_2. **a**, Schematic of an example post-growth ABC/ACB morphology with two domain walls. **b-c**, Domain wall motion and reaction on flat SiO_2 leading to ABC domain growth.

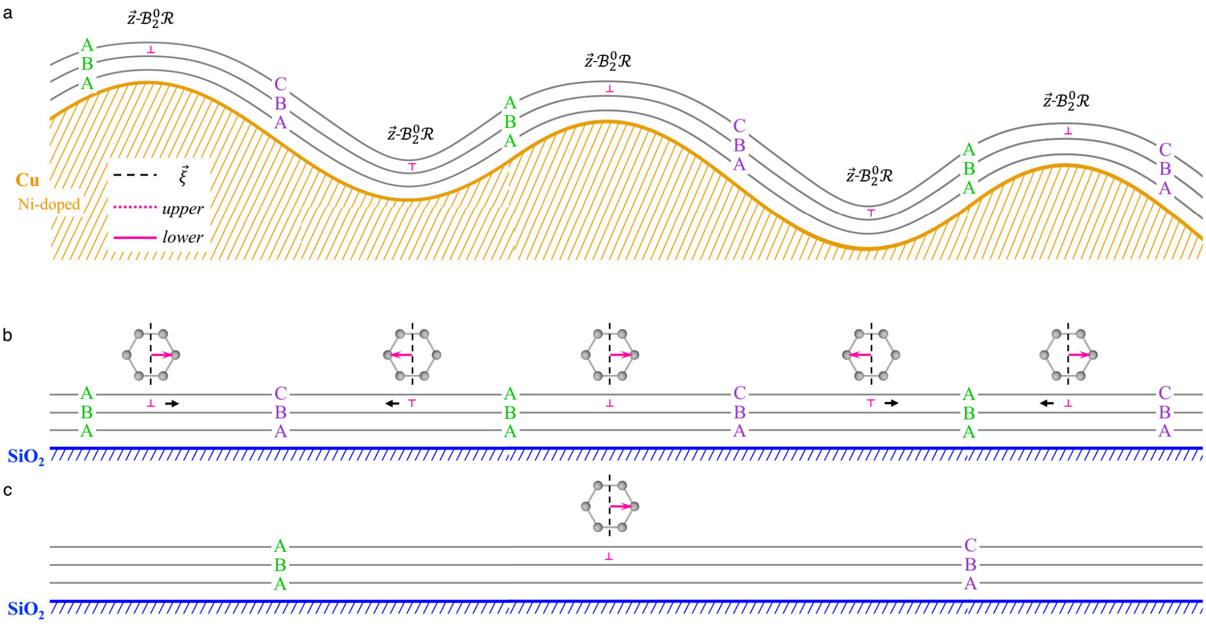

Supplementary Figure 18. Coarsening of domain morphologies in ABA/ABC flakes transferred from the corrugated nickel-doped copper substrate to flat SiO_2. **a**, Schematic of an example post-growth ABA/ABC morphology with five domain walls. **b-c**, Domain wall motion and reaction on flat SiO_2 leading to larger ABA and ABC domains.

Supplementary Note 3: Correlation between surface corrugations on the growth substrate and TLG domain walls

To examine the correlation between surface corrugations and TLG domain walls, we patterned markers onto a CVD-grown (and PMMA-coated) graphene + substrate sample using e-beam lithography followed by O_2 plasma etching, as shown in **Supplementary Fig. 19a**. HCl etching (10% aqueous solution) was then used to create markers at the same locations on the substrate surface (**Supplementary Fig. 19a**). The PMMA coating preserves the substrate surface morphologies during the marker etching steps. The TLG was then delaminated from the marked substrate via the bubbling transfer method[22] and transferred to a TEM copper grid (**Supplementary Fig. 19a**). DFTEM images were then obtained, allowing domain wall and stacking morphologies to be precisely mapped back to AFM topography and amplitude images at the same location where growth occurred (**Supplementary Figs. 19b-c, 20**).

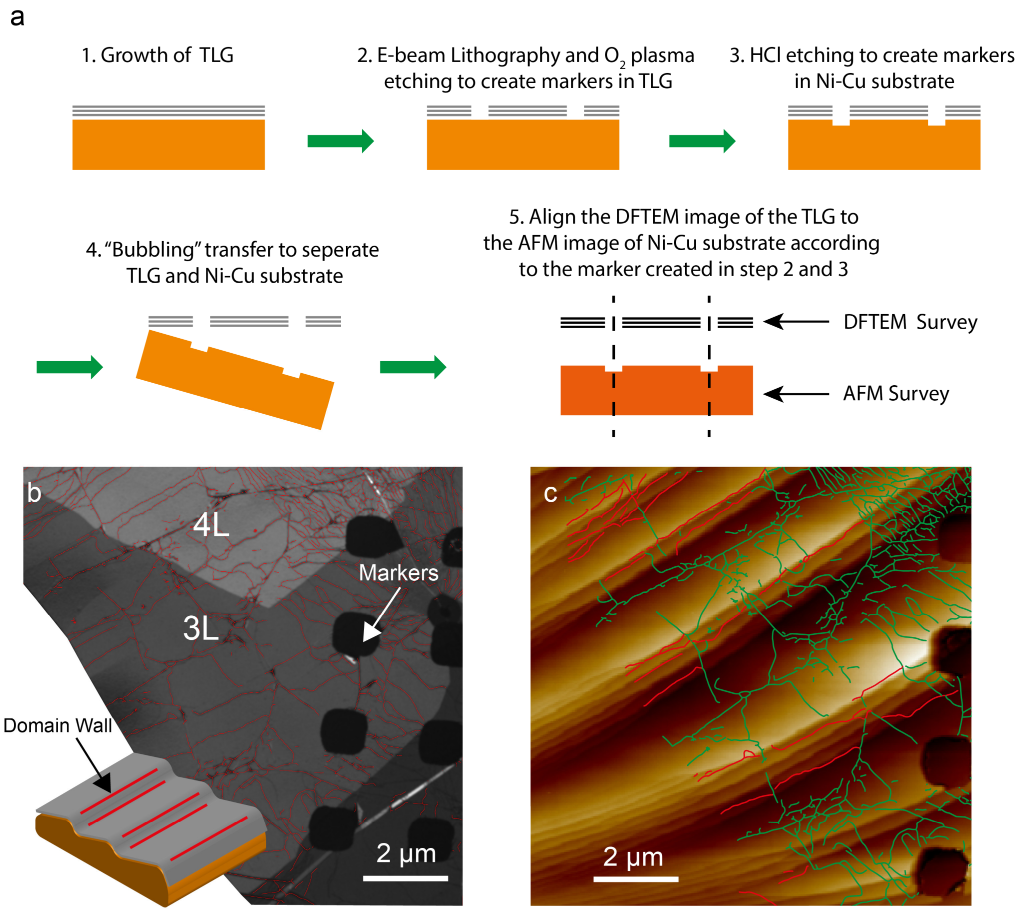

Supplementary Figure 19. a, Schematic showing the fabrication of alignment marks on TLG and Ni-Cu substrate. The as-grown TLG on Ni-Cu substrate was patterned using e-beam lithography, followed by O_2 plasma etching and HCl etching. Bubbling transfer[22] was then carried out to transfer TLG onto a Cu grid for DFTEM investigations, followed by AFM investigations on the corresponding area of the Ni-Cu substrate to correlate the substrate corrugations with the TLG stacking domain wall morphology. **b**, Second order DFTEM image showing domain walls highlighted by red lines. The marker arrays are used to map the domain walls to their original growth substrate. **c**, Mapping of domains walls onto AFM topography images. Red lines denote walls strongly correlated with substrate corrugations, green lines denote walls not associated with corrugations (very likely due to strains induced during marking, bubbling transfer, and/or the graphene etching processes).

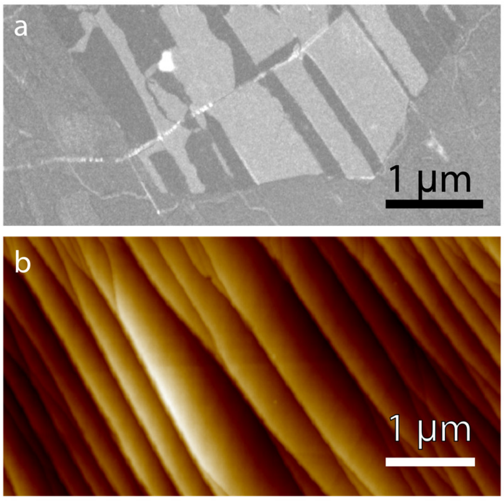

Supplementary Figure 20. a, First order DFTEM image of the TLG flake in **Fig. 3f**. **b**, AFM topography image of the growth substrate in **Fig. 3f**. Scale bar: 70 nm.

Supplementary Note 4: Room temperature transport measurements on ABC- and ABA-TLG

As noted in the main text, after transfer the TLG material had relatively large domains (~ 1 μm) of ABC and ABA stacked material that could be contacted individually for electronic measurements. As shown in **Supplementary Fig. 21a**, single-domain FET devices were created in a dual-gate configuration using 45 nm thick HfO_2 deposited by atomic layer deposition as the top-gate dielectric and 250 nm thick thermally grown SiO_2 as the bottom-gate dielectric. The measurement was carried out in a probe station with a bias voltage of 15 mV applied by a Keithley 2400 source meter. The top-gate/bottom-gate voltage was applied using a Keithley 6517A/Keithley 6487 voltage source.

We measured the resistance between different pairs of electrodes that contacted regions with different layer stacking while varying the top and bottom gate voltages. **Supplementary Fig. 21b** shows resistance versus top-gate voltage (R-V_{TG}) curves between electrodes 1 and 2, which were in contact with an ABC-TLG domain. Each R-V_{TG} curve was measured with a fixed back-gate voltage (V_{BG}) ranging from -50 V to 100 V in steps of 10 V, and V_{TG} was swept continuously. The existence of an electric-field tunable energy band gap in CVD ABC-TLG is consistent with the increase in on/off ratio as the strength of the out-of-plane electric field is increased. No such

increase was observed for the ABA-TLG FET between electrodes 2 and 3 (**Supplementary Fig. 21c**). The tunable band gap of ABC-TLG at room temperature (293 K) agrees well with the low temperature (1.8 K) transport results (main manuscript).

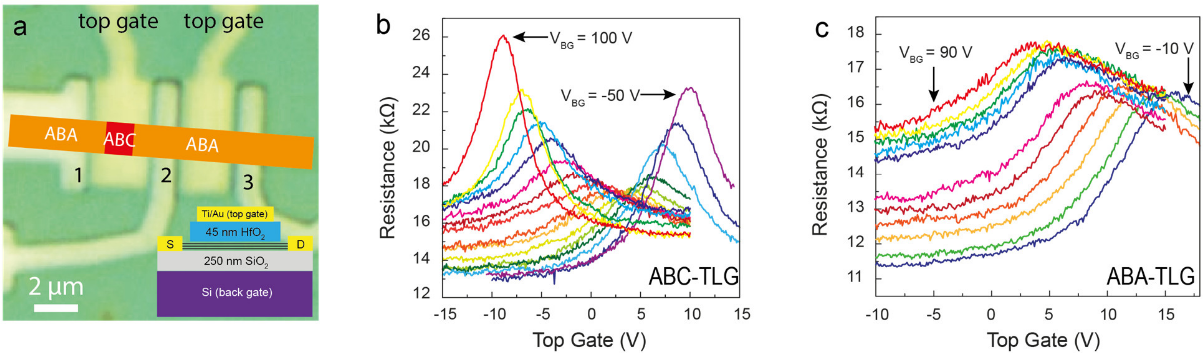

Supplementary Figure 21. Electrical transport measurements at room temperature. **a**, Optical image of the dual-gated TLG device. The red area represents the ABC-TLG channel and the orange area is the ABA-TLG channel. Three gold electrodes are marked by Arabic numerals. The inset shows the cross-section of the device. **b**, R-V_{TG} characteristics of CVD ABC-TLG (electrodes 1 and 2). Each curve was measured with a fixed V_{BG} ranging from -50 V to 100 V in steps of 10 V, indicated by different colors. The on/off ratios increase with V_{BG} due to the band gap opening of ABC-TLG. **c**, R-V_{TG} characteristics of CVD ABA-TLG (electrodes 2 and 3). Each curve was measured with a fixed V_{BG} ranging from -10 V to 90 V in 10 V steps.

Supplementary Note 5: Substrate curvature engineering for controllable ABC-TLG synthesis

Here we further demonstrate the potential practical significance of our topography engineering concept for the large-area synthesis of high quality TLG with controlled stacking. We propose a previously overlooked physical mechanism driving corrugation coarsening during graphene growth and incorporate this mechanism into a time-dependent model of corrugation and stacking configuration evolution. The model is consistent with experimental evidence which demonstrates that surface topography and stacking configurations can be controlled by varying the growth time.

Physics and phenomenology of corrugation formation and coarsening

Corrugation formation or step edge bunching on crystal surfaces has been studied for several decades in relation to, e.g., heteroepitaxial thin film growth. Surface steps produce elastic fields and interact with each other through these fields[23]. The mutual interactions between steps are intrinsically repulsive when the surface is unstressed, with the repulsive pairwise force decaying as α_2/L^3, where α_2 is a constant and L is the distance between steps[24]. Therefore, unstressed films typically adopt a relatively flat surface with a uniformly distributed array of steps. When the film is under a stress σ, an attractive force of the form α_1/L emerges between steps, where $\alpha_1 = \sigma^2 h_0^2/ML$, h_0 is the step height, and M is an elastic constant. This drives steps to bunch into groups with each step at the minimum energy separation $L_0 = \sqrt{\alpha_2/\alpha_1}$ from its neighbors. Stressed films with surface steps therefore exhibit step bunching at temperatures high enough to facilitate significant adatom diffusion, resulting in a coarsely corrugated surface.

Our growth substrates typically exhibited nearly flat topographies pre-growth. Corrugations were observed on both Cu-Ni and Cu substrates after graphene growth and only on regions of the substrate covered by graphene (**Supplementary Fig. 22**). Corrugation coarseness (amplitude and wavelength) was larger below BLG flakes than below MLG flakes (**Supplementary Fig. 22a**).

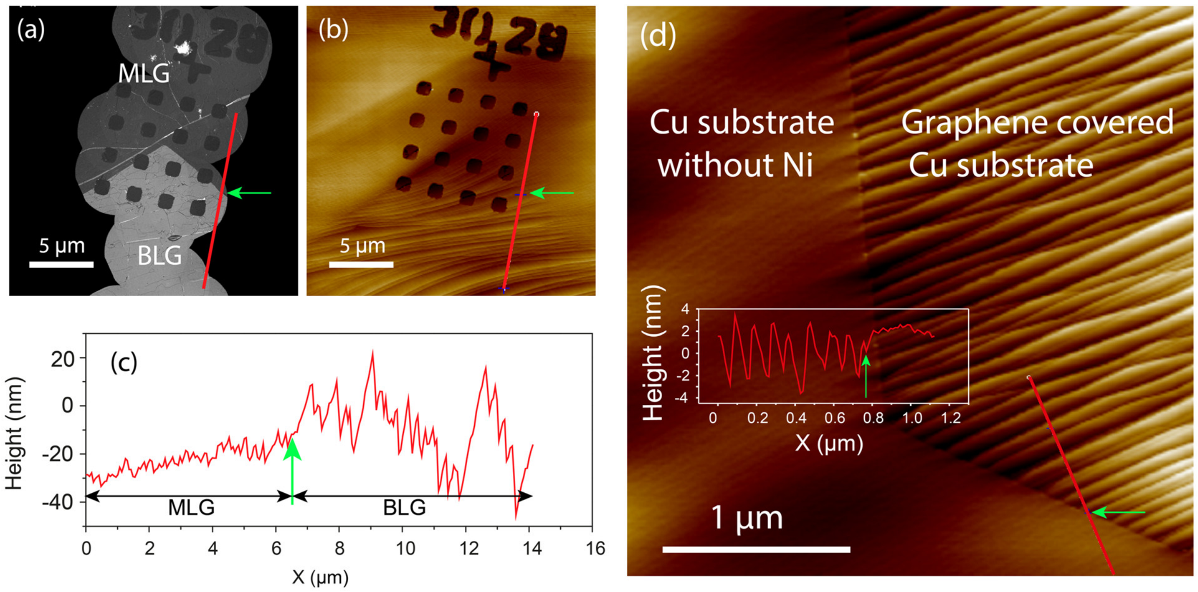

Supplementary Figure 22. Corrugation formation on Ni-Cu and Cu substrates, observed after graphene growth. **a**, DFTEM image showing MLG and BLG regions grown on a Ni-Cu substrate and **b**-**c**, corresponding AFM measurements of substrate corrugations (AFM height scale: 218 nm). The linescan profile in **c** (red lines in **a** and **b**) shows coarser corrugations below BLG than MLG, consistent with previous results[25-28] **d**, AFM image of a pure Cu substrate (without Ni) after graphene growth. Height (contrast) scale: 16 nm. Corrugation formation occurs in the graphene covered region, as was observed for Cu-Ni substrates.

These results indicate that graphene flakes impart stresses onto the substrate surface and are consistent with previous studies[25-28]. Corrugations have been widely observed on growth substrates following CVD synthesis of graphene and other 2D materials. They typically have a quasi-1D, lamellar topography with spacings λ and heights h that range from $10 \lesssim \lambda \lesssim 500$ nm and $1 \lesssim h \lesssim 50$ nm. Their origin has been debated and means to reliably control their morphology have not been identified. The stresses driving corrugation formation could arise from bulk lattice misfit strain between graphene and Cu, which is ~2.5–6%, depending on Cu surface crystallography, temperature, and Cu alloying (typically, graphene is stretched, Cu is compressed). Moiré patterns (localized slip and possibly out-of-plane bulging) widely observed in graphene overlayers may indicate that some portion of any bulk lattice misfit stress is relieved. Thermal expansion mismatch between graphene and Cu has also been proposed to generate stresses that drive corrugation formation during cooling[25-27].

A recent experimental and computational study confirmed that substrate corrugations of the type seen here form and coarsen beneath graphene *during* growth, not during cooling[28]. They proposed that the driving force for corrugation formation and coarsening is the reduction in graphene bending energy obtained by merging many small pre-existing surface steps into fewer larger ones with less curvature. They also proposed that the process is kinetically enabled by rapid

Cu adatom diffusion beneath the growing graphene flakes. This mechanism is consistent with the central experimental observations that corrugations become coarser with higher growth temperatures (increased adatom mobility) and with increasing number of graphene layers (increased driving force), as observed here.

However, we find that the bending energy of graphene is insufficient to drive step edge merging. The molecular dynamics (MD) simulations reported in Ref. [28] of Cu step edge merging below a graphene overlayer exhibit a ~3 eV per unit cell decrease in total energy upon merging several unit steps into one large step. The bending energy of the graphene sheet should decrease by at least this amount if reduction of bending energy is the driving force. We compute the total bending energy of the reported graphene topography profiles $w(x)$ before and after step edge merging as

$$E_{\text{bend}} = \frac{\kappa Ly}{2}\int_0^{Lx} [w''(x)]^2 \, dx,$$

where $\kappa \approx 1$ eV is the bending stiffness of graphene, Lx and Ly are the end-to-end lengths of the graphene sheet, and $w''(x) = d^2w(x)/dx^2$ is the curvature profile of the sheet. The resulting energy change accounts for only ~1% of the total observed ~3 eV per unit cell decrease, indicating that bending energy is not the primary driving force for step edge merging.

We propose that the previously overlooked effect of in-plane interlayer disregistry/dislocation energy between graphene and the substrate at step edges or corrugations (as depicted in **Fig. 3b**) is a more significant driving force in this process. Interlayer disregistry/dislocation energy is significantly reduced by merging many small corrugations into fewer large corrugations. It is easiest to consider the two limiting cases of this effect. If the interlayer interaction is strong enough to enforce perfect atomic registry in the presence of curvature ($\kappa_0 < \kappa_c$), then the curvature is translated directly and entirely into in-plane strain (plus a very small graphene bending energy). The stored energy is proportional to the in-plane modulus,

$$E_{\text{disreg}} = \frac{E^{2\text{D}}}{2}\int_0^{Lx} \left[\frac{d_3 w''(x)}{1 + d_3 w''(x)}\right]^2 dx,$$

where $E^{2\text{D}}$ is the 2D Young's modulus of graphene and d_3 is the Cu-graphene interlayer separation (see also **Fig. 3b**). Following the calculation above for the MD graphene topography profiles $w(x)$ in Ref. [28], the resulting energy change upon merging several unit steps into one large step accounts for ~1–2 eV of the total ~3 eV per unit cell decrease.

However, as discussed in the main manuscript, when the substrate curvature is sufficiently large for a given interlayer interaction energy ($\kappa_0 < \kappa_c$), interlayer dislocation formation (localized slip that relieves the disregistry strain) becomes energetically preferable to maintaining registry. In this configuration, much of the in-plane strain is relaxed and the energy becomes localized in narrow dislocation cores (plus a small contribution from long range strain fields, see definition of E_{disloc} in **Methods**). If we consider the limit in which interlayer dislocations exist at each step, a similar or larger energy decrease in the MD system is obtained due to reduction of the total dislocation energy E_{disloc}. An optimal combination of E_{disreg} and E_{disloc} reduction should be realized in general, and these estimates indicate that this combination can readily account for the measured ~3 eV per unit cell decrease.

We therefore propose that an array of initially small step edges below a graphene overlayer is driven to merge and coarsen to reduce the total amount of interlayer disregistry/dislocation energy. This excess energy drives the redistribution of Cu adatoms into a surface topography that reduces $E_{\text{disreg}} + E_{\text{disloc}}$ as much as possible. Since $E_{\text{disreg}} + E_{\text{disloc}}$ scales approximately linearly with the number of graphene layers, we expect the rate of coarsening to increase with the number of layers, consistent with our results shown in **Supplementary Fig. 22** and with previous experimental[25-28] and simulation results[28]. We also see from this analysis that as graphene drives corrugation coarsening, it drives changes in its own internal domain wall structure through alterations in the balance of E_{disreg} and E_{disloc}.

A second potentially significant (and related) driving force is the reduction of total lattice misfit energy between substrate and graphene *via* preferential growth of low energy crystallographic surface domains. Some Cu surfaces provide a better lattice match to graphene than others, and if these surfaces can be exposed by Cu adatom redistribution during growth, then their total area may increase with time. This could occur through growth of preexisting domains or nucleation and growth of new domains and would tend to produce large, flat surface domains separated by increasingly sharp or faceted transition regions, as observed in our system.

Physical description and control of curvature for large-area synthesis of ABC-TLG

Here we incorporate substrate topography kinetics driven by graphene overlayers into our continuum model for domain wall energetics by postulating a time-dependent expression for the maximum topographic curvature κ_0. This allows examination of changes in the preferred domain wall morphologies as corrugations evolve during growth.

Our experimental results show that corrugation amplitude and wavelength increase with time and/or number of graphene layers. This is consistent with elastically-driven 1D step bunching *via* surface adatom diffusion[23,29,30]. We also find that the maximum curvature (at extrema) generally increases with time (**Fig. 3g**) in a type of faceting process. Our data are consistent with a $\kappa_0 \sim t^{1/3}$ behavior, which may indicate that the dominant mechanism is diffusive transport of an approximately conserved number of surface steps. We postulate here accordingly that $\kappa_0(t) \approx \kappa_0(0) + Kt^{1/3}$, where K is a constant kinetic prefactor that depends on step edge mobility $M_{\text{s}}(T)$, density ρ_{s}, and height h_0. These parameters are dependent on surface crystal orientation, indicating that topography and thus ABC yield will vary with orientation. This is confirmed experimentally in **Supplementary Fig. 6**, where different surface orientations are shown to produce different topographies within a single TLG flake. The resulting effect on ABC yield is consistent with expectations based on our model. Further study will be required to establish explicit connections between specific surfaces and coarsening parameters and to assess the dominant surface kinetic mechanisms for different systems in detail.

Substitution of $\kappa_0(t)$ above into our model for domain wall energetics provides a description of minimum energy domain wall configurations versus growth time (t). The upper horizontal axis in **Fig. 3e** shows qualitatively how geometry (corrugation curvature κ_0 in this case) is translated to growth time in this description, for a specific choice of $\kappa_0(0)$ and K (presently unknown). Flakes evolve during growth from near the left side of the plot (small corrugations with low maximum curvature) toward the right side (larger corrugations with higher maximum curvature). The preferred domain wall types thus change during growth, trending from domain wall-free ABA states to states with domain walls and increasing ABC fraction, to states with domain walls and decreasing ABC fraction. This indicates that the ABC fraction should tend to

first increase and then decrease for long growth times. Surfaces that do not facet with time as those here are expected to result in maximum curvatures that decrease with time, i.e., a reversal of the present trends.

Graphene itself drives substrate corrugation coarsening during CVD growth to relieve step / curvature-induced interlayer disregistry/dislocation energy (and possibly lattice misfit strain). This coarsening feeds back into the structure of the growing graphene flake, dictating which domain walls and stacking configurations are energetically preferred as a function of growth time / corrugation topography. The area fraction of ABC phase is thus controllable by tuning growth time, temperature, substrate surface crystal orientation, and/or initial topography to maintain ABC-stabilizing topographies during as much of the growth process as possible.

To demonstrate control of curvature for large-area synthesis of ABC-TLG, we experimentally show that substrate curvature and TLG stacking configurations are tunable through the CVD growth time. TLG samples were grown on Ni-Cu substrates for different durations of time, followed by the fabrication of alignment marks as described in **Supplementary Fig. 19**. The ABC ratios within TLG flakes and the corresponding substrate curvatures beneath the TLG were investigated. As shown in **Fig. 3g**, the average peak corrugation curvature κ_0 of the TLG-covered regions evolves systematically with the growth time, increasing from ~7.0 $\times 10^6$ m^{-1} to ~1.4 $\times 10^7$ m^{-1} as the growth time increases from 1 hour to 6 hours. Based on the modeling predictions shown in **Fig. 3e**, we expect the resulting TLG flakes to vary from low ABC yield to approximately maximal ABC yield. This is in good agreement with the ABC yields shown in **Fig. 3g (**also see **Supplementary Figs. 23** and **24**). ABC-TLG ratios increase significantly as κ_0 increases from 7.0 $\times 10^6$ m^{-1} (1 hour growth) to 1.1 $\times 10^7$ m^{-1} (3 hour growth) and the ABA to ABC boundary is crossed. Longer growth times and larger curvatures lead to decreased ABC ratios, consistent with our expectation of a finite range of optimal curvatures. The modest quantitative disagreement on the position of the ABC to ABA boundary is not surprising given the approximations employed and the experimental uncertainties. The above results demonstrate the capability of practical control of substrate curvature for large-area synthesis of ABC-TLG based on our approach. More precise control and higher yields of ABC-TLG can be achieved with further exploration of other parameters, i.e., growth temperature, substrate chemistry, and substrate crystallography.

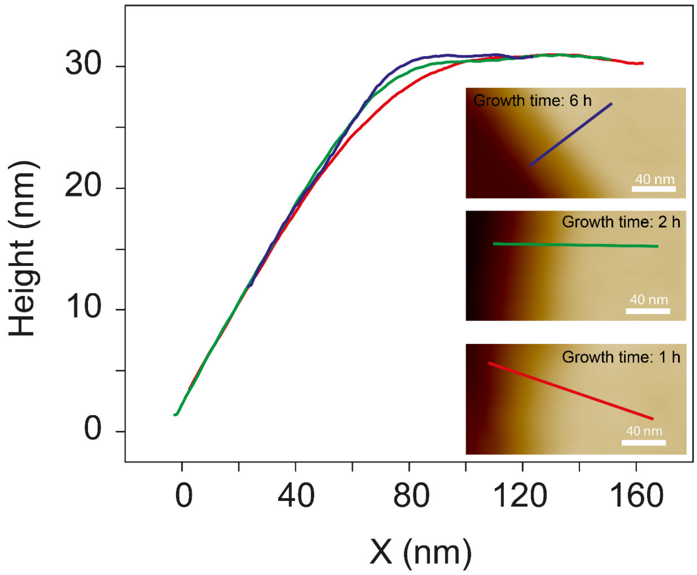

Supplementary Figure 23. Typical AFM linescan profiles over substrate regions where TLG was grown

for the times indicated. The peak curvatures increase with growth time. Statistical averages are shown in the top panel of **Fig. 3g**.

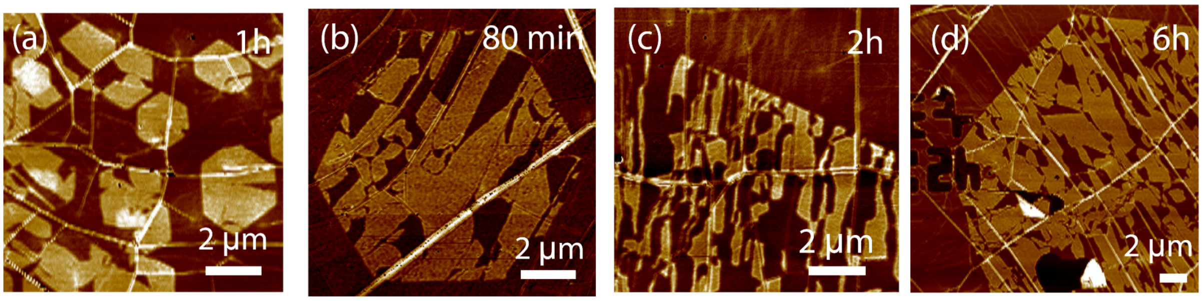

Supplementary Figure 24. IR-SNOM images of typical ABC-TLG flakes synthesized with different growth times, the 3 hour growth is shown in **Supplementary Fig. 7**.

This physical view of disregistry-driven substrate corrugation formation and coarsening is applicable to other vdW systems. It provides the essential elements of a strategy for designing growth procedures that incorporate dynamic substrate engineering to optimize material quality and controllably grow vdW materials with targeted domain wall and stacking configurations. Sets of optimal conditions for, *e.g.*, ABC stabilization, must be determined in terms of growth time, temperature, substrate surface crystal orientation, and initial topography. This framework will also be an essential component in understanding and systematically improving the types and quality of CVD-grown multilayer materials. As seen in **Supplementary Fig. 25**, both 4-layer and 5-layer graphene grown by CVD show structures with differing stacking orders, which we believe is driven by the same underlying physics.

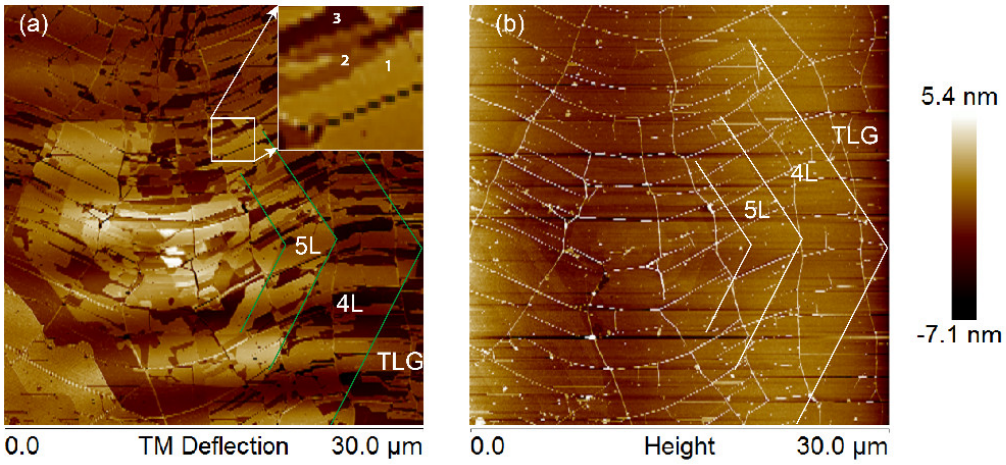

Supplementary Figure 25. (a) IR-SNOM and (b) AFM images of multilayer graphene transferred onto a SiO_2 substrate. The green lines in panel (a) separate graphene flakes with different numbers of layers. The inset shows that the five-layer region has more than two stacking orders as labeled by the Arabic numerals.

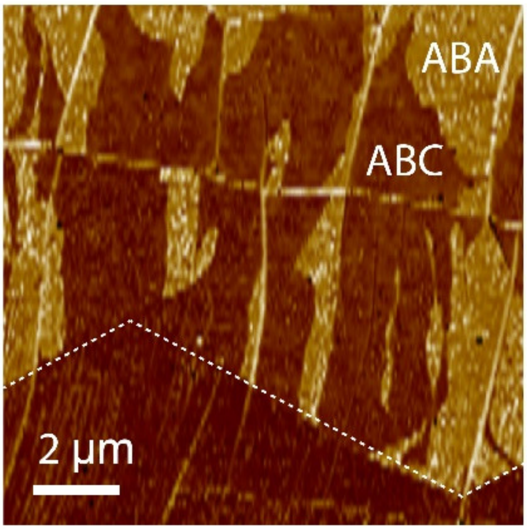

Supplementary Figure 26. IR-SNOM image of CVD grown ABC-TLG after transfer onto an hBN substrate, showing stabilization of large-area ABC-TLG on hBN, which occurs only very rarely for exfoliated material[31]. The dashed lines indicate the boundaries between bilayer graphene and TLG.

Supplementary References

1. Brown, L. *et al.* Twinning and Twisting of Tri- and Bilayer Graphene. *Nano Lett.* **12**, 1609-1615 (2012).
2. Han, G. H. *et al.* Continuous Growth of Hexagonal Graphene and Boron Nitride In-Plane Heterostructures by Atmospheric Pressure Chemical Vapor Deposition. *ACS Nano* **7**, 10129-10138 (2013).
3. Luo, Z., Kim, S., Kawamoto, N., Rappe, A. M. & Johnson, A. T. C. Growth Mechanism of Hexagonal-Shape Graphene Flakes with Zigzag Edges. *ACS Nano* **5**, 9154-9160 (2011).
4. Chanier, T. & Henrard, L. From carbon atom to graphene on Cu(111): an ab-initio study. *The European Physical Journal B* **88**, 5 (2015).
5. Giovannetti, G. *et al.* Doping Graphene with Metal Contacts. *Phys. Rev. Lett.* **101**, 026803 (2008).
6. Gebhardt, J., Viñes, F. & Görling, A. Influence of the surface dipole layer and Pauli repulsion on band energies and doping in graphene adsorbed on metal surfaces. *Phys. Rev. B* **86**, 195431 (2012).
7. Hamada, I. & Otani, M. Comparative van der Waals density-functional study of graphene on metal surfaces. *Physical Review B* **82**, 153412 (2010).
8. C., S. R., L., S. J. & V., C. P. Graphene–Graphene Interactions: Friction, Superlubricity, and Exfoliation. *Adv. Mater.* **30**, 1705791 (2018).
9. Feng, X., Kwon, S., Park, J. Y. & Salmeron, M. Superlubric Sliding of Graphene Nanoflakes on Graphene. *ACS Nano* **7**, 1718-1724 (2013).
10. Gao, Z. *et al.* Crystalline Bilayer Graphene with Preferential Stacking from Ni–Cu Gradient Alloy. *ACS Nano* **12**, 2275-2282 (2018).
11. Slonczewski, J. C. & Weiss, P. R. Band Structure of Graphite. *Physical Review* **109**, 272-279 (1958).

12. McClure, J. W. Band Structure of Graphite and de Haas-van Alphen Effect. *Physical Review* **108**, 612-618 (1957).
13. Zhang, W. *et al.* Molecular adsorption induces the transformation of rhombohedral- to Bernal-stacking order in trilayer graphene. *Nature Communications* **4**, 2074 (2013).
14. Charlier, J. C., Gonze, X. & Michenaud, J. P. First-principles study of the stacking effect on the electronic properties of graphite(s). *Carbon* **32**, 289-299 (1994).
15. Aoki, M. & Amawashi, H. Dependence of band structures on stacking and field in layered graphene. *Solid State Commun.* **142**, 123-127 (2007).
16. Tkatchenko, A., DiStasio, R. A., Car, R. & Scheffler, M. Accurate and Efficient Method for Many-Body van der Waals Interactions. *Phys. Rev. Lett.* **108**, 236402 (2012).
17. Krukau, A. V., Vydrov, O. A., Izmaylov, A. F. & Scuseria, G. E. Influence of the exchange screening parameter on the performance of screened hybrid functionals. *The Journal of Chemical Physics* **125**, 224106 (2006).
18. Lee, C., Wei, X., Kysar, J. W. & Hone, J. Measurement of the Elastic Properties and Intrinsic Strength of Monolayer Graphene. *Science* **321**, 385 (2008).
19. Al-Jishi, R. & Dresselhaus, G. Lattice-dynamical model for alkali-metal-graphite intercalation compounds. *Phys. Rev. B* **26**, 4523-4538 (1982).
20. Dai, S., Xiang, Y. & Srolovitz, D. J. Structure and energetics of interlayer dislocations in bilayer graphene. *Physical Review B* **93**, 085410 (2016).
21. Butz, B. *et al.* Dislocations in bilayer graphene. *Nature* **505**, 533-537 (2014).
22. Gao, Z. *et al.* Scalable Production of Sensor Arrays Based on High-Mobility Hybrid Graphene Field Effect Transistors. *ACS Appl. Mater. Interfaces* **8**, 27546-27552 (2016).
23. Tersoff, J., Phang, Y. H., Zhang, Z. & Lagally, M. G. Step-Bunching Instability of Vicinal Surfaces under Stress. *Phys. Rev. Lett.* **75**, 2730-2733 (1995).
24. Marchenko, V. & Parshin, A. Y. Elastic properties of crystal surfaces. *Sov. Phys. JETP* **52**, 129 (1980).
25. Tian, J. *et al.* Graphene Induced Surface Reconstruction of Cu. *Nano Lett.* **12**, 3893-3899 (2012).
26. Kim, D. W., Lee, J., Kim, S. J., Jeon, S. & Jung, H.-T. The effects of the crystalline orientation of Cu domains on the formation of nanoripple arrays in CVD-grown graphene on Cu. *Journal of Materials Chemistry C* **1**, 7819-7824 (2013).
27. Kang, J. H. *et al.* Strain Relaxation of Graphene Layers by Cu Surface Roughening. *Nano Lett.* **16**, 5993-5998 (2016).
28. Yi, D. *et al.* What Drives Metal-Surface Step Bunching in Graphene Chemical Vapor Deposition? *Phys. Rev. Lett.* **120**, 246101 (2018).
29. Léonard, F. & Tersoff, J. Competing step instabilities at surfaces under stress. *Appl. Phys. Lett.* **83**, 72-74 (2003).
30. Bisschop, J. & Dysthe, D. K. Instabilities and Coarsening of Stressed Crystal Surfaces in Aqueous Solution. *Phys. Rev. Lett.* **96**, 146103 (2006).
31. Chen, G. *et al.* Evidence of a gate-tunable Mott insulator in a trilayer graphene moiré superlattice. *Nat. Phys.* **15**, 237-241 (2019).
